# Supplementary figures and images for: Emerging fungal pathogen of an invasive grass: Implications for competition with native plant species
Source: PLoS One. 2021 Mar 1;16(3):e0237894. doi: 10.1371/journal.pone.0237894 (PMC7920361; doi:10.1371/journal.pone.0237894)

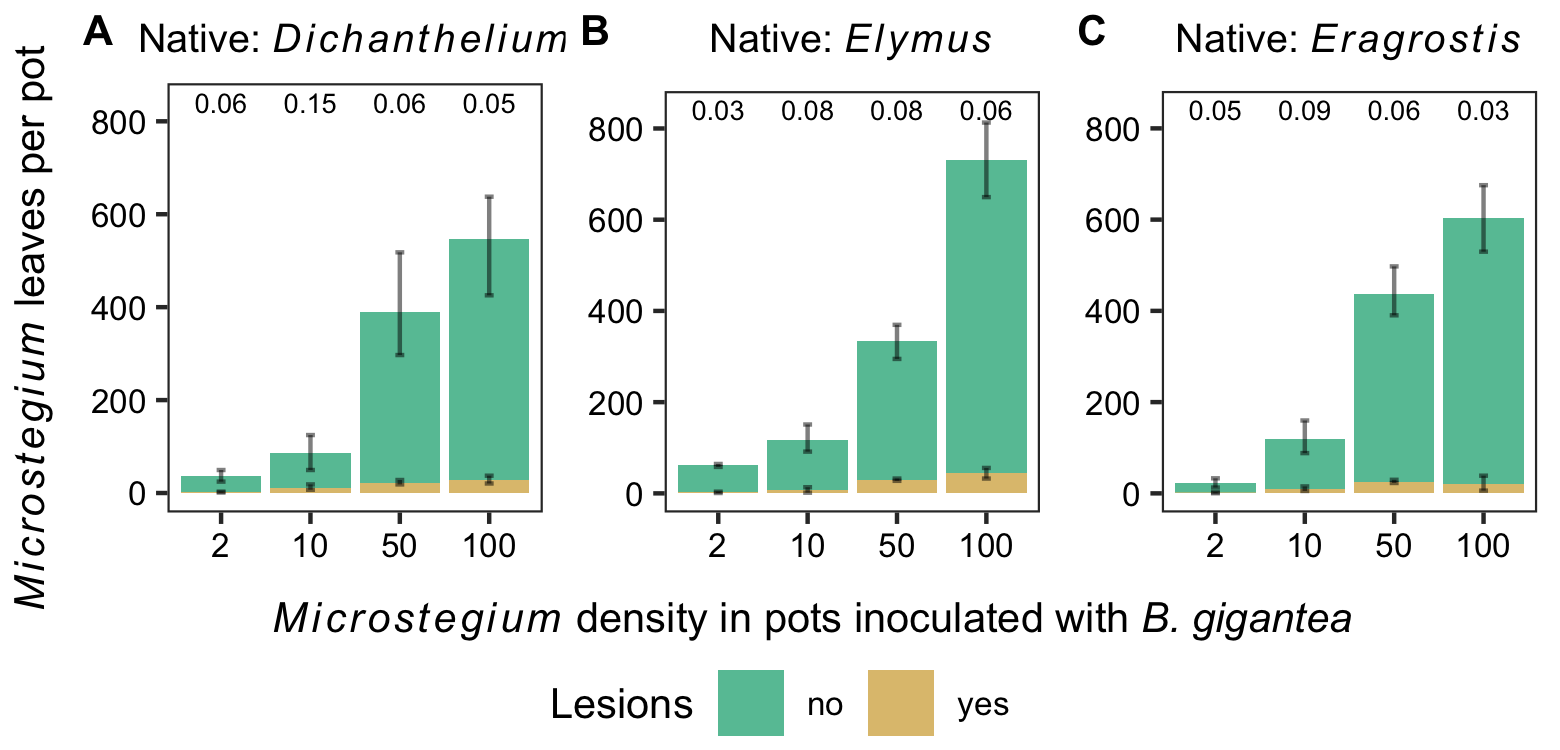

Supplement: S1 Fig — The estimated number of Microstegium leaves with lesions across the Microstegium density gradient following B. gigantea inoculation when grown in the presence of (A) Dichanthelium, (B) Elymus, and (C) Eragrostis (mean ± 95% confidence intervals). All leaves with lesions were counted and the total number leaves per pot were estimated by counting the number of leaves on up to three plants per pot. (TIF) [file pone.0237894.s001.tif]
