# Supplementary material for: Emerging fungal pathogen of an invasive grass: Implications for competition with native plant species
Source: PLoS One. 2021 Mar 1;16(3):e0237894. doi: 10.1371/journal.pone.0237894 (PMC7920361; doi:10.1371/journal.pone.0237894)
Supplement: S1 Table — Raw data collected from an experiment at BONWR in which fungicide or water (control) were added to plots with ten planting treatments. Leaves of Microstegium vimineum (Mv) and Elymus virginicus (Ev) were assessed for visible eyespots and B. gigantea conidiophores using microscopy. (DOCX) [file pone.0237894.s002.docx]

**S1 Table. *Bipolaris gigantea* identification on field-collected leaves.** Raw data collected from an experiment at BONWR in which fungicide or water (control) were added to plots with ten planting treatments. Leaves of *Microstegium vimineum* (Mv) and *Elymus virginicus* (Ev) were assessed for visible eyespots and *B. gigantea* conidiophores using microscopy.

| Year | Month | Site | Plot | Treatment | Species | Eyespots | *B. gigantea* |
| --- | --- | --- | --- | --- | --- | --- | --- |
| 2018 | July | D1 | 1 | fungicide | Mv | 0 | 1 |
| 2018 | July | D1 | 2 | fungicide | Mv | 1 | 1 |
| 2018 | July | D1 | 3 | fungicide | Mv | 0 | 0 |
| 2018 | July | D1 | 4 | fungicide | Mv | 1 | 1 |
| 2018 | July | D1 | 5 | fungicide | Mv | 1 | 1 |
| 2018 | July | D1 | 6 | fungicide | Mv | 0 | 0 |
| 2018 | July | D1 | 7 | fungicide | Mv | 1 | 1 |
| 2018 | July | D1 | 8 | fungicide | Mv | 1 | 1 |
| 2018 | July | D1 | 9 | fungicide | Mv | 0 | 1 |
| 2018 | July | D1 | 10 | fungicide | Mv | 1 | 0 |
| 2018 | July | D1 | 1 | water | Mv | 1 | 0 |
| 2018 | July | D1 | 2 | water | Mv | 1 | 1 |
| 2018 | July | D1 | 3 | water | Mv | 1 | 1 |
| 2018 | July | D1 | 4 | water | Mv | 1 | 0 |
| 2018 | July | D1 | 5 | water | Mv | 0 | 1 |
| 2018 | July | D1 | 6 | water | Mv | 1 | 1 |
| 2018 | July | D1 | 7 | water | Mv | 1 | 1 |
| 2018 | July | D1 | 8 | water | Mv | 1 | 1 |
| 2018 | July | D1 | 9 | water | Mv | 1 | 1 |
| 2018 | July | D1 | 10 | water | Mv | 1 | 1 |
| 2018 | July | D1 | 1 | fungicide | Ev | 0 | 0 |
| 2018 | July | D1 | 2 | fungicide | Ev | 1 | 1 |
| 2018 | July | D1 | 3 | fungicide | Ev | 0 | 0 |
| 2018 | July | D1 | 4 | fungicide | Ev | 0 | 0 |
| 2018 | July | D1 | 5 | fungicide | Ev | 0 | 0 |
| 2018 | July | D1 | 6 | fungicide | Ev | 0 | 0 |
| 2018 | July | D1 | 8 | fungicide | Ev | 0 | 0 |
| 2018 | July | D1 | 9 | fungicide | Ev | 0 | 0 |
| 2018 | July | D1 | 10 | fungicide | Ev | 0 | 0 |
| 2018 | July | D1 | 1 | water | Ev | 0 | 0 |
| 2018 | July | D1 | 2 | water | Ev | 0 | 0 |
| 2018 | July | D1 | 3 | water | Ev | 0 | 0 |
| 2018 | July | D1 | 4 | water | Ev | 0 | 0 |
| 2018 | July | D1 | 5 | water | Ev | 0 | 0 |
| 2018 | July | D1 | 6 | water | Ev | 0 | 0 |
| 2018 | July | D1 | 7 | water | Ev | 0 | 0 |
| 2018 | July | D1 | 8 | water | Ev | 0 | 0 |
| 2018 | July | D1 | 9 | water | Ev | 0 | 0 |
| 2018 | July | D1 | 10 | water | Ev | 1 | 1 |
| 2018 | July | D2 | 1 | fungicide | Mv | 1 | 1 |
| 2018 | July | D2 | 2 | fungicide | Mv | 0 | 0 |
| 2018 | July | D2 | 3 | fungicide | Mv | 1 | 1 |
| 2018 | July | D2 | 4 | fungicide | Mv | 1 | 0 |
| 2018 | July | D2 | 5 | fungicide | Mv | 1 | 1 |
| 2018 | July | D2 | 6 | fungicide | Mv | 0 | 1 |
| 2018 | July | D2 | 7 | fungicide | Mv | 1 | 0 |
| 2018 | July | D2 | 8 | fungicide | Mv | 1 | 1 |
| 2018 | July | D2 | 9 | fungicide | Mv | 1 | 1 |
| 2018 | July | D2 | 10 | fungicide | Mv | 1 | 0 |
| 2018 | July | D2 | 1 | water | Mv | 0 | 0 |
| 2018 | July | D2 | 2 | water | Mv | 1 | 1 |
| 2018 | July | D2 | 3 | water | Mv | 1 | 1 |
| 2018 | July | D2 | 4 | water | Mv | 1 | 1 |
| 2018 | July | D2 | 5 | water | Mv | 1 | 1 |
| 2018 | July | D2 | 6 | water | Mv | 1 | 1 |
| 2018 | July | D2 | 7 | water | Mv | 1 | 1 |
| 2018 | July | D2 | 8 | water | Mv | 1 | 1 |
| 2018 | July | D2 | 9 | water | Mv | 1 | 1 |
| 2018 | July | D2 | 10 | water | Mv | 1 | 1 |
| 2018 | July | D2 | 1 | fungicide | Ev | 0 | 0 |
| 2018 | July | D2 | 3 | fungicide | Ev | 0 | 0 |
| 2018 | July | D2 | 4 | fungicide | Ev | 0 | 0 |
| 2018 | July | D2 | 5 | fungicide | Ev | 1 | 0 |
| 2018 | July | D2 | 6 | fungicide | Ev | 0 | 0 |
| 2018 | July | D2 | 8 | fungicide | Ev | 1 | 0 |
| 2018 | July | D2 | 9 | fungicide | Ev | 0 | 0 |
| 2018 | July | D2 | 10 | fungicide | Ev | 0 | 0 |
| 2018 | July | D2 | 3 | water | Ev | 0 | 0 |
| 2018 | July | D2 | 4 | water | Ev | 0 | 0 |
| 2018 | July | D2 | 5 | water | Ev | 0 | 0 |
| 2018 | July | D2 | 6 | water | Ev | 1 | 0 |
| 2018 | July | D2 | 7 | water | Ev | 1 | 0 |
| 2018 | July | D2 | 10 | water | Ev | 1 | 0 |
| 2018 | July | D3 | 1 | fungicide | Mv | 0 | 0 |
| 2018 | July | D3 | 2 | fungicide | Mv | 1 | 0 |
| 2018 | July | D3 | 3 | fungicide | Mv | 0 | 0 |
| 2018 | July | D3 | 4 | fungicide | Mv | 0 | 0 |
| 2018 | July | D3 | 5 | fungicide | Mv | 0 | 0 |
| 2018 | July | D3 | 6 | fungicide | Mv | 1 | 0 |
| 2018 | July | D3 | 7 | fungicide | Mv | 1 | 1 |
| 2018 | July | D3 | 8 | fungicide | Mv | 0 | 1 |
| 2018 | July | D3 | 9 | fungicide | Mv | 0 | 0 |
| 2018 | July | D3 | 10 | fungicide | Mv | 0 | 0 |
| 2018 | July | D3 | 1 | water | Mv | 1 | 1 |
| 2018 | July | D3 | 2 | water | Mv | 1 | 1 |
| 2018 | July | D3 | 3 | water | Mv | 1 | 1 |
| 2018 | July | D3 | 4 | water | Mv | 1 | 1 |
| 2018 | July | D3 | 5 | water | Mv | 1 | 1 |
| 2018 | July | D3 | 6 | water | Mv | 1 | 0 |
| 2018 | July | D3 | 7 | water | Mv | 0 | 0 |
| 2018 | July | D3 | 8 | water | Mv | 1 | 0 |
| 2018 | July | D3 | 9 | water | Mv | 1 | 0 |
| 2018 | July | D3 | 10 | water | Mv | 0 | 0 |
| 2018 | July | D3 | 1 | fungicide | Ev | 0 | 0 |
| 2018 | July | D3 | 2 | fungicide | Ev | 0 | 0 |
| 2018 | July | D3 | 3 | fungicide | Ev | 0 | 0 |
| 2018 | July | D3 | 4 | fungicide | Ev | 0 | 0 |
| 2018 | July | D3 | 5 | fungicide | Ev | 0 | 0 |
| 2018 | July | D3 | 6 | fungicide | Ev | 0 | 0 |
| 2018 | July | D3 | 7 | fungicide | Ev | 0 | 0 |
| 2018 | July | D3 | 8 | fungicide | Ev | 0 | 0 |
| 2018 | July | D3 | 9 | fungicide | Ev | 1 | 0 |
| 2018 | July | D3 | 10 | fungicide | Ev | 1 | 0 |
| 2018 | July | D3 | 1 | water | Ev | 0 | 0 |
| 2018 | July | D3 | 2 | water | Ev | 0 | 0 |
| 2018 | July | D3 | 3 | water | Ev | 0 | 0 |
| 2018 | July | D3 | 4 | water | Ev | 0 | 0 |
| 2018 | July | D3 | 5 | water | Ev | 0 | 0 |
| 2018 | July | D3 | 6 | water | Ev | 0 | 0 |
| 2018 | July | D3 | 7 | water | Ev | 0 | 0 |
| 2018 | July | D3 | 8 | water | Ev | 0 | 0 |
| 2018 | July | D3 | 9 | water | Ev | 0 | 0 |
| 2018 | July | D3 | 10 | water | Ev | 1 | 0 |
| 2018 | July | D4 | 1 | fungicide | Mv | 0 | 1 |
| 2018 | July | D4 | 2 | fungicide | Mv | 1 | 1 |
| 2018 | July | D4 | 3 | fungicide | Mv | 1 | 1 |
| 2018 | July | D4 | 4 | fungicide | Mv | 1 | 1 |
| 2018 | July | D4 | 5 | fungicide | Mv | 1 | 0 |
| 2018 | July | D4 | 7 | fungicide | Mv | 1 | 1 |
| 2018 | July | D4 | 9 | fungicide | Mv | 1 | 1 |
| 2018 | July | D4 | 1 | water | Mv | 1 | 1 |
| 2018 | July | D4 | 2 | water | Mv | 0 | 1 |
| 2018 | July | D4 | 3 | water | Mv | 1 | 1 |
| 2018 | July | D4 | 4 | water | Mv | 1 | 0 |
| 2018 | July | D4 | 5 | water | Mv | 0 | 0 |
| 2018 | July | D4 | 6 | water | Mv | 1 | 1 |
| 2018 | July | D4 | 7 | water | Mv | 1 | 1 |
| 2018 | July | D4 | 8 | water | Mv | 0 | 1 |
| 2018 | July | D4 | 9 | water | Mv | 1 | 1 |
| 2018 | July | D4 | 10 | water | Mv | 1 | 1 |
| 2018 | July | D4 | 1 | fungicide | Ev | 0 | 0 |
| 2018 | July | D4 | 2 | fungicide | Ev | 0 | 0 |
| 2018 | July | D4 | 3 | fungicide | Ev | 0 | 0 |
| 2018 | July | D4 | 4 | fungicide | Ev | 0 | 0 |
| 2018 | July | D4 | 5 | fungicide | Ev | 0 | 0 |
| 2018 | July | D4 | 6 | fungicide | Ev | 0 | 0 |
| 2018 | July | D4 | 7 | fungicide | Ev | 1 | 1 |
| 2018 | July | D4 | 9 | fungicide | Ev | 0 | 0 |
| 2018 | July | D4 | 1 | water | Ev | 0 | 0 |
| 2018 | July | D4 | 2 | water | Ev | 0 | 0 |
| 2018 | July | D4 | 3 | water | Ev | 0 | 0 |
| 2018 | July | D4 | 4 | water | Ev | 0 | 0 |
| 2018 | July | D4 | 5 | water | Ev | 0 | 0 |
| 2018 | July | D4 | 6 | water | Ev | 0 | 0 |
| 2018 | July | D4 | 7 | water | Ev | 0 | 0 |
| 2018 | July | D4 | 8 | water | Ev | 0 | 0 |
| 2018 | July | D4 | 9 | water | Ev | 0 | 0 |
| 2018 | July | D4 | 10 | water | Ev | 0 | 0 |
| 2018 | late August | D1 | 8 | fungicide | Mv | 1 | 1 |
| 2018 | late August | D1 | 10 | fungicide | Mv | 1 | 1 |
| 2018 | late August | D1 | 2 | water | Mv | 1 | 1 |
| 2018 | late August | D1 | 6 | water | Mv | 1 | 1 |
| 2018 | late August | D1 | 7 | water | Mv | 1 | 1 |
| 2018 | late August | D1 | 9 | water | Mv | 1 | 1 |
| 2018 | late August | D1 | 2 | fungicide | Ev | 1 | 1 |
| 2018 | late August | D1 | 3 | fungicide | Ev | 0 | 0 |
| 2018 | late August | D1 | 4 | fungicide | Ev | 0 | 0 |
| 2018 | late August | D1 | 6 | fungicide | Ev | 0 | 0 |
| 2018 | late August | D1 | 8 | fungicide | Ev | 1 | 1 |
| 2018 | late August | D1 | 9 | fungicide | Ev | 1 | 0 |
| 2018 | late August | D1 | 10 | fungicide | Ev | 0 | 0 |
| 2018 | late August | D1 | 1 | water | Ev | 1 | 0 |
| 2018 | late August | D1 | 2 | water | Ev | 1 | 0 |
| 2018 | late August | D1 | 3 | water | Ev | 1 | 1 |
| 2018 | late August | D1 | 6 | water | Ev | 1 | 1 |
| 2018 | late August | D1 | 7 | water | Ev | 1 | 1 |
| 2018 | late August | D1 | 9 | water | Ev | 1 | 1 |
| 2018 | late August | D1 | 10 | water | Ev | 1 | 1 |
| 2018 | late August | D2 | 1 | fungicide | Mv | 1 | 1 |
| 2018 | late August | D2 | 3 | fungicide | Mv | 1 | 1 |
| 2018 | late August | D2 | 5 | fungicide | Mv | 1 | 1 |
| 2018 | late August | D2 | 6 | fungicide | Mv | 1 | 1 |
| 2018 | late August | D2 | 8 | fungicide | Mv | 1 | 1 |
| 2018 | late August | D2 | 2 | water | Mv | 1 | 1 |
| 2018 | late August | D2 | 3 | water | Mv | 1 | 1 |
| 2018 | late August | D2 | 4 | water | Mv | 1 | 1 |
| 2018 | late August | D2 | 5 | fungicide | Ev | 1 | 0 |
| 2018 | late August | D2 | 6 | fungicide | Ev | 0 | 0 |
| 2018 | late August | D2 | 8 | fungicide | Ev | 1 | 1 |
| 2018 | late August | D2 | 3 | water | Ev | 0 | 0 |
| 2018 | late August | D2 | 6 | water | Ev | 1 | 1 |
| 2018 | late August | D2 | 7 | water | Ev | 1 | 1 |
| 2018 | late August | D2 | 8 | water | Ev | 1 | 1 |
| 2018 | late August | D2 | 9 | water | Ev | 0 | 0 |
| 2018 | late August | D2 | 10 | water | Ev | 1 | 1 |
| 2018 | late August | D3 | 1 | fungicide | Mv | 1 | 1 |
| 2018 | late August | D3 | 4 | fungicide | Mv | 1 | 1 |
| 2018 | late August | D3 | 6 | fungicide | Mv | 1 | 1 |
| 2018 | late August | D3 | 9 | fungicide | Mv | 1 | 1 |
| 2018 | late August | D3 | 2 | water | Mv | 1 | 1 |
| 2018 | late August | D3 | 3 | water | Mv | 1 | 1 |
| 2018 | late August | D3 | 4 | water | Mv | 1 | 1 |
| 2018 | late August | D3 | 6 | water | Mv | 1 | 1 |
| 2018 | late August | D3 | 7 | water | Mv | 1 | 1 |
| 2018 | late August | D3 | 8 | water | Mv | 1 | 1 |
| 2018 | late August | D3 | 1 | fungicide | Ev | 0 | 0 |
| 2018 | late August | D3 | 2 | fungicide | Ev | 0 | 0 |
| 2018 | late August | D3 | 4 | fungicide | Ev | 0 | 0 |
| 2018 | late August | D3 | 7 | fungicide | Ev | 1 | 1 |
| 2018 | late August | D3 | 9 | fungicide | Ev | 0 | 0 |
| 2018 | late August | D3 | 10 | fungicide | Ev | 1 | 0 |
| 2018 | late August | D3 | 2 | water | Ev | 1 | 1 |
| 2018 | late August | D3 | 3 | water | Ev | 1 | 0 |
| 2018 | late August | D3 | 6 | water | Ev | 0 | 0 |
| 2018 | late August | D3 | 7 | water | Ev | 0 | 0 |
| 2018 | late August | D3 | 9 | water | Ev | 0 | 0 |
| 2018 | late August | D3 | 10 | water | Ev | 0 | 0 |
| 2018 | late August | D4 | 2 | water | Mv | 1 | 1 |
| 2018 | late August | D4 | 10 | water | Mv | 1 | 1 |
| 2018 | late August | D4 | 3 | fungicide | Ev | 0 | 0 |
| 2018 | late August | D4 | 5 | fungicide | Ev | 0 | 0 |
| 2018 | late August | D4 | 6 | fungicide | Ev | 0 | 0 |
| 2018 | late August | D4 | 9 | fungicide | Ev | 0 | 0 |
| 2018 | late August | D4 | 2 | water | Ev | 0 | 0 |
| 2018 | late August | D4 | 3 | water | Ev | 0 | 0 |
| 2018 | late August | D4 | 5 | water | Ev | 1 | 0 |
| 2018 | late August | D4 | 8 | water | Ev | 1 | 1 |
| 2018 | late August | D4 | 10 | water | Ev | 1 | 1 |
| 2018 | September | D1 | 4 | fungicide | Mv | 1 | 1 |
| 2018 | September | D1 | 5 | fungicide | Mv | 1 | 1 |
| 2018 | September | D1 | 6 | fungicide | Mv | 1 | 1 |
| 2018 | September | D1 | 7 | fungicide | Mv | 1 | 1 |
| 2018 | September | D1 | 8 | fungicide | Mv | 1 | 1 |
| 2018 | September | D1 | 9 | fungicide | Mv | 1 | 1 |
| 2018 | September | D1 | 10 | fungicide | Mv | 1 | 1 |
| 2018 | September | D1 | 1 | water | Mv | 1 | 1 |
| 2018 | September | D1 | 2 | water | Mv | 1 | 1 |
| 2018 | September | D1 | 4 | water | Mv | 1 | 1 |
| 2018 | September | D1 | 6 | water | Mv | 1 | 1 |
| 2018 | September | D1 | 7 | water | Mv | 1 | 1 |
| 2018 | September | D1 | 9 | water | Mv | 1 | 1 |
| 2018 | September | D1 | 10 | water | Mv | 1 | 1 |
| 2018 | September | D1 | 2 | fungicide | Ev | 1 | 0 |
| 2018 | September | D1 | 3 | fungicide | Ev | 0 | 0 |
| 2018 | September | D1 | 4 | fungicide | Ev | 0 | 0 |
| 2018 | September | D1 | 6 | fungicide | Ev | 0 | 0 |
| 2018 | September | D1 | 8 | fungicide | Ev | 1 | 0 |
| 2018 | September | D1 | 10 | fungicide | Ev | 0 | 0 |
| 2018 | September | D1 | 3 | water | Ev | 1 | 0 |
| 2018 | September | D1 | 6 | water | Ev | 0 | 0 |
| 2018 | September | D1 | 7 | water | Ev | 1 | 0 |
| 2018 | September | D1 | 8 | water | Ev | 0 | 0 |
| 2018 | September | D1 | 9 | water | Ev | 0 | 0 |
| 2018 | September | D2 | 1 | fungicide | Mv | 1 | 1 |
| 2018 | September | D2 | 2 | fungicide | Mv | 1 | 0 |
| 2018 | September | D2 | 3 | fungicide | Mv | 1 | 1 |
| 2018 | September | D2 | 4 | fungicide | Mv | 1 | 1 |
| 2018 | September | D2 | 5 | fungicide | Mv | 1 | 1 |
| 2018 | September | D2 | 6 | fungicide | Mv | 1 | 1 |
| 2018 | September | D2 | 7 | fungicide | Mv | 1 | 1 |
| 2018 | September | D2 | 8 | fungicide | Mv | 1 | 1 |
| 2018 | September | D2 | 9 | fungicide | Mv | 1 | 1 |
| 2018 | September | D2 | 1 | water | Mv | 1 | 1 |
| 2018 | September | D2 | 2 | water | Mv | 1 | 0 |
| 2018 | September | D2 | 3 | water | Mv | 1 | 0 |
| 2018 | September | D2 | 4 | water | Mv | 1 | 0 |
| 2018 | September | D2 | 5 | water | Mv | 1 | 0 |
| 2018 | September | D2 | 6 | water | Mv | 1 | 0 |
| 2018 | September | D2 | 7 | water | Mv | 1 | 1 |
| 2018 | September | D2 | 8 | water | Mv | 1 | 0 |
| 2018 | September | D2 | 9 | water | Mv | 1 | 0 |
| 2018 | September | D2 | 10 | water | Mv | 1 | 1 |
| 2018 | September | D2 | 5 | fungicide | Ev | 1 | 0 |
| 2018 | September | D2 | 6 | fungicide | Ev | 0 | 0 |
| 2018 | September | D2 | 8 | fungicide | Ev | 0 | 0 |
| 2018 | September | D2 | 3 | water | Ev | 0 | 0 |
| 2018 | September | D2 | 6 | water | Ev | 0 | 0 |
| 2018 | September | D2 | 7 | water | Ev | 1 | 0 |
| 2018 | September | D2 | 9 | water | Ev | 1 | 0 |
| 2018 | September | D2 | 10 | water | Ev | 0 | 1 |
| 2018 | September | D3 | 1 | fungicide | Mv | 1 | 0 |
| 2018 | September | D3 | 2 | fungicide | Mv | 1 | 1 |
| 2018 | September | D3 | 3 | fungicide | Mv | 1 | 0 |
| 2018 | September | D3 | 4 | fungicide | Mv | 1 | 0 |
| 2018 | September | D3 | 5 | fungicide | Mv | 1 | 1 |
| 2018 | September | D3 | 6 | fungicide | Mv | 1 | 0 |
| 2018 | September | D3 | 7 | fungicide | Mv | 1 | 0 |
| 2018 | September | D3 | 8 | fungicide | Mv | 1 | 0 |
| 2018 | September | D3 | 9 | fungicide | Mv | 1 | 1 |
| 2018 | September | D3 | 10 | fungicide | Mv | 1 | 0 |
| 2018 | September | D3 | 1 | water | Mv | 1 | 0 |
| 2018 | September | D3 | 2 | water | Mv | 1 | 1 |
| 2018 | September | D3 | 3 | water | Mv | 1 | 0 |
| 2018 | September | D3 | 4 | water | Mv | 1 | 1 |
| 2018 | September | D3 | 5 | water | Mv | 1 | 0 |
| 2018 | September | D3 | 6 | water | Mv | 1 | 1 |
| 2018 | September | D3 | 7 | water | Mv | 1 | 0 |
| 2018 | September | D3 | 8 | water | Mv | 1 | 0 |
| 2018 | September | D3 | 9 | water | Mv | 1 | 1 |
| 2018 | September | D3 | 10 | water | Mv | 1 | 0 |
| 2018 | September | D4 | 1 | fungicide | Mv | 1 | 1 |
| 2018 | September | D4 | 2 | fungicide | Mv | 1 | 0 |
| 2018 | September | D4 | 3 | fungicide | Mv | 1 | 0 |
| 2018 | September | D4 | 4 | fungicide | Mv | 1 | 1 |
| 2018 | September | D4 | 5 | fungicide | Mv | 1 | 0 |
| 2018 | September | D4 | 6 | fungicide | Mv | 1 | 1 |
| 2018 | September | D4 | 7 | fungicide | Mv | 1 | 0 |
| 2018 | September | D4 | 8 | fungicide | Mv | 0 | 0 |
| 2018 | September | D4 | 9 | fungicide | Mv | 1 | 0 |
| 2018 | September | D4 | 10 | fungicide | Mv | 0 | 0 |
| 2018 | September | D4 | 1 | water | Mv | 1 | 0 |
| 2018 | September | D4 | 2 | water | Mv | 1 | 1 |
| 2018 | September | D4 | 3 | water | Mv | 1 | 0 |
| 2018 | September | D4 | 4 | water | Mv | 1 | 1 |
| 2018 | September | D4 | 5 | water | Mv | 1 | 0 |
| 2018 | September | D4 | 6 | water | Mv | 1 | 0 |
| 2018 | September | D4 | 7 | water | Mv | 1 | 1 |
| 2018 | September | D4 | 8 | water | Mv | 1 | 0 |
| 2018 | September | D4 | 9 | water | Mv | 1 | 0 |
| 2018 | September | D4 | 10 | water | Mv | 1 | 0 |
| 2018 | September | D4 | 3 | fungicide | Ev | 1 | 0 |
| 2018 | September | D4 | 5 | fungicide | Ev | 0 | 0 |
| 2018 | September | D4 | 6 | fungicide | Ev | 0 | 0 |
| 2018 | September | D4 | 9 | fungicide | Ev | 0 | 0 |
| 2018 | September | D4 | 2 | water | Ev | 0 | 0 |
| 2018 | September | D4 | 3 | water | Ev | 0 | 0 |
| 2018 | September | D4 | 5 | water | Ev | 0 | 0 |
| 2018 | September | D4 | 8 | water | Ev | 0 | 0 |
| 2018 | September | D4 | 10 | water | Ev | 0 | 0 |
| 2019 | July | D1 | 1 | removal | Mv | 1 | 1 |
| 2019 | July | D1 | 1 | control | Mv | 1 | 1 |
| 2019 | July | D1 | 1 | addition | Mv | 1 | 1 |
| 2019 | July | D1 | 2 | removal | Mv | 1 | 0 |
| 2019 | July | D1 | 2 | control | Mv | 1 | 1 |
| 2019 | July | D1 | 2 | addition | Mv | 1 | 1 |
| 2019 | July | D3 | 1 | removal | Mv | 1 | 1 |
| 2019 | July | D3 | 1 | control | Mv | 1 | 1 |
| 2019 | July | D3 | 1 | addition | Mv | 1 | 1 |
| 2019 | July | D3 | 2 | removal | Mv | 1 | 1 |
| 2019 | July | D3 | 2 | control | Mv | 1 | 1 |
| 2019 | July | D3 | 2 | addition | Mv | 1 | 1 |
| 2019 | July | D1 | 1 | water | Mv | 1 | 0 |
| 2019 | July | D1 | 2 | water | Mv | 1 | 1 |
| 2019 | July | D1 | 3 | water | Mv | 1 | 1 |
| 2019 | July | D1 | 4 | water | Mv | 1 | 0 |
| 2019 | July | D1 | 5 | water | Mv | 0 | 0 |
| 2019 | July | D1 | 6 | water | Mv | 1 | 0 |
| 2019 | July | D1 | 7 | water | Mv | 1 | 0 |
| 2019 | July | D1 | 8 | water | Mv | 1 | 1 |
| 2019 | July | D1 | 9 | water | Mv | 1 | 0 |
| 2019 | July | D1 | 10 | water | Mv | 1 | 1 |
| 2019 | July | D1 | 2 | fungicide | Mv | 1 | 1 |
| 2019 | July | D1 | 3 | fungicide | Mv | 1 | 0 |
| 2019 | July | D1 | 4 | fungicide | Mv | 1 | 0 |
| 2019 | July | D1 | 5 | fungicide | Mv | 1 | 0 |
| 2019 | July | D1 | 6 | fungicide | Mv | 0 | 0 |
| 2019 | July | D1 | 7 | fungicide | Mv | 1 | 0 |
| 2019 | July | D1 | 8 | fungicide | Mv | 1 | 1 |
| 2019 | July | D1 | 9 | fungicide | Mv | 1 | 0 |
| 2019 | July | D1 | 10 | fungicide | Mv | 1 | 0 |
| 2019 | July | D2 | 1 | water | Mv | 0 | 0 |
| 2019 | July | D2 | 2 | water | Mv | 1 | 1 |
| 2019 | July | D2 | 3 | water | Mv | 1 | 0 |
| 2019 | July | D2 | 4 | water | Mv | 1 | 1 |
| 2019 | July | D2 | 5 | water | Mv | 1 | 1 |
| 2019 | July | D2 | 6 | water | Mv | 1 | 1 |
| 2019 | July | D2 | 7 | water | Mv | 1 | 1 |
| 2019 | July | D2 | 8 | water | Mv | 1 | 1 |
| 2019 | July | D2 | 9 | water | Mv | 1 | 1 |
| 2019 | July | D2 | 10 | water | Mv | 1 | 1 |
| 2019 | July | D2 | 1 | fungicide | Mv | 1 | 1 |
| 2019 | July | D2 | 2 | fungicide | Mv | 1 | 0 |
| 2019 | July | D2 | 3 | fungicide | Mv | 1 | 1 |
| 2019 | July | D2 | 4 | fungicide | Mv | 1 | 0 |
| 2019 | July | D2 | 5 | fungicide | Mv | 1 | 1 |
| 2019 | July | D2 | 6 | fungicide | Mv | 1 | 1 |
| 2019 | July | D2 | 7 | fungicide | Mv | 0 | 0 |
| 2019 | July | D2 | 8 | fungicide | Mv | 1 | 1 |
| 2019 | July | D2 | 9 | fungicide | Mv | 1 | 1 |
| 2019 | July | D2 | 10 | fungicide | Mv | 0 | 0 |
| 2019 | July | D3 | 1 | water | Mv | 1 | 1 |
| 2019 | July | D3 | 2 | water | Mv | 1 | 1 |
| 2019 | July | D3 | 3 | water | Mv | 1 | 0 |
| 2019 | July | D3 | 4 | water | Mv | 1 | 1 |
| 2019 | July | D3 | 5 | water | Mv | 1 | 1 |
| 2019 | July | D3 | 6 | water | Mv | 1 | 1 |
| 2019 | July | D3 | 7 | water | Mv | 1 | 0 |
| 2019 | July | D3 | 8 | water | Mv | 1 | 0 |
| 2019 | July | D3 | 9 | water | Mv | 1 | 1 |
| 2019 | July | D3 | 10 | water | Mv | 1 | 1 |
| 2019 | July | D3 | 1 | fungicide | Mv | 1 | 1 |
| 2019 | July | D3 | 2 | fungicide | Mv | 1 | 0 |
| 2019 | July | D3 | 3 | fungicide | Mv | 1 | 1 |
| 2019 | July | D3 | 4 | fungicide | Mv | 1 | 1 |
| 2019 | July | D3 | 5 | fungicide | Mv | 1 | 0 |
| 2019 | July | D3 | 6 | fungicide | Mv | 1 | 1 |
| 2019 | July | D3 | 7 | fungicide | Mv | 1 | 1 |
| 2019 | July | D3 | 8 | fungicide | Mv | 1 | 1 |
| 2019 | July | D3 | 9 | fungicide | Mv | 1 | 1 |
| 2019 | July | D3 | 10 | fungicide | Mv | 1 | 0 |
| 2019 | July | D4 | 1 | water | Mv | 0 | 0 |
| 2019 | July | D4 | 2 | water | Mv | 1 | 1 |
| 2019 | July | D4 | 3 | water | Mv | 1 | 1 |
| 2019 | July | D4 | 4 | water | Mv | 1 | 0 |
| 2019 | July | D4 | 5 | water | Mv | 1 | 0 |
| 2019 | July | D4 | 6 | water | Mv | 1 | 0 |
| 2019 | July | D4 | 7 | water | Mv | 1 | 1 |
| 2019 | July | D4 | 8 | water | Mv | 1 | 0 |
| 2019 | July | D4 | 9 | water | Mv | 1 | 0 |
| 2019 | July | D4 | 10 | water | Mv | 1 | 1 |
| 2019 | July | D4 | 1 | fungicide | Mv | 1 | 1 |
| 2019 | July | D4 | 2 | fungicide | Mv | 1 | 1 |
| 2019 | July | D4 | 3 | fungicide | Mv | 1 | 1 |
| 2019 | July | D4 | 4 | fungicide | Mv | 1 | 0 |
| 2019 | July | D4 | 5 | fungicide | Mv | 1 | 0 |
| 2019 | July | D4 | 6 | fungicide | Mv | 1 | 0 |
| 2019 | July | D4 | 7 | fungicide | Mv | 1 | 1 |
| 2019 | July | D4 | 8 | fungicide | Mv | 1 | 0 |
| 2019 | July | D4 | 9 | fungicide | Mv | 1 | 0 |
| 2019 | July | D4 | 10 | fungicide | Mv | 1 | 0 |
| 2019 | early August | D1 | 3 | water | Ev | 1 | 1 |
| 2019 | early August | D1 | 8 | water | Ev | 1 | 1 |
| 2019 | early August | D1 | 8 | fungicide | Ev | 1 | 1 |
| 2019 | early August | D2 | 2 | water | Ev | 1 | 1 |
| 2019 | early August | D2 | 3 | water | Ev | 0 | 0 |
| 2019 | early August | D2 | 4 | water | Ev | 0 | 0 |
| 2019 | early August | D2 | 6 | water | Ev | 1 | 1 |
| 2019 | early August | D2 | 7 | water | Ev | 1 | 1 |
| 2019 | early August | D2 | 8 | water | Ev | 1 | 1 |
| 2019 | early August | D2 | 10 | water | Ev | 1 | 1 |
| 2019 | early August | D3 | 4 | fungicide | Ev | 1 | 1 |
| 2019 | early August | D4 | 1 | water | Ev | 1 | 0 |
| 2019 | early August | D4 | 2 | water | Ev | 1 | 0 |
| 2019 | early August | D4 | 3 | water | Ev | 1 | 1 |
| 2019 | early August | D4 | 4 | water | Ev | 0 | 0 |
| 2019 | early August | D4 | 7 | water | Ev | 1 | 1 |
| 2019 | early August | D4 | 8 | water | Ev | 1 | 0 |
| 2019 | early August | D4 | 9 | water | Ev | 1 | 0 |
| 2019 | early August | D4 | 10 | water | Ev | 1 | 0 |
| 2019 | early August | D4 | 1 | fungicide | Ev | 0 | 0 |
| 2019 | early August | D4 | 2 | fungicide | Ev | 1 | 0 |
| 2019 | early August | D4 | 3 | fungicide | Ev | 0 | 0 |
| 2019 | early August | D4 | 5 | fungicide | Ev | 1 | 1 |
| 2019 | early August | D4 | 6 | fungicide | Ev | 1 | 0 |
| 2019 | early August | D4 | 7 | fungicide | Ev | 1 | 0 |
| 2019 | early August | D4 | 8 | fungicide | Ev | 1 | 0 |
| 2019 | early August | D4 | 9 | fungicide | Ev | 1 | 0 |
| 2019 | early August | D4 | 10 | fungicide | Ev | 1 | 0 |
