# Supplementary material for: Emerging fungal pathogen of an invasive grass: Implications for competition with native plant species
Source: PLoS One. 2021 Mar 1;16(3):e0237894. doi: 10.1371/journal.pone.0237894 (PMC7920361; doi:10.1371/journal.pone.0237894)
Supplement: S2 Table — Raw data collected from Microstegium in an experiment at BONWR in which the total number of leaves per stem and the number of leaves with at least two foliar lesions per stem were recorded. The plots included were sprayed monthly with water a control for fungicide (not included). (DOCX) [file pone.0237894.s003.docx]

**S2 Table. *Microstegium vimineum* infection incidence in the field.** Raw data collected from *Microstegium* in an experiment at BONWR in which the total number of leaves per stem and the number of leaves with at least two foliar lesions per stem were recorded. The plots included were sprayed monthly with water a control for fungicide (not included).

| Year | Month | Site | Plot | Plant ID | Total leaves | Leaves with lesions |
| --- | --- | --- | --- | --- | --- | --- |
| 2018 | July | D1 | 10 | 1 | 5 | 5 |
| 2018 | July | D1 | 10 | 2 | 5 | 3 |
| 2018 | July | D1 | 10 | 3 | 10 | 6 |
| 2018 | July | D1 | 1 | 1 | 7 | 2 |
| 2018 | July | D1 | 1 | 2 | 7 | 0 |
| 2018 | July | D1 | 1 | 3 | 8 | 1 |
| 2018 | July | D1 | 2 | 1 | 7 | 5 |
| 2018 | July | D1 | 2 | 2 | 5 | 4 |
| 2018 | July | D1 | 2 | 3 | 6 | 4 |
| 2018 | July | D1 | 3 | 1 | 8 | 7 |
| 2018 | July | D1 | 3 | 2 | 5 | 5 |
| 2018 | July | D1 | 3 | 3 | 5 | 4 |
| 2018 | July | D1 | 4 | 1 | 5 | 0 |
| 2018 | July | D1 | 4 | 3 | 5 | 0 |
| 2018 | July | D1 | 5 | 2 | 3 | 0 |
| 2018 | July | D1 | 5 | 3 | 6 | 0 |
| 2018 | July | D1 | 6 | 1 | 5 | 2 |
| 2018 | July | D1 | 6 | 2 | 4 | 2 |
| 2018 | July | D1 | 6 | 3 | 8 | 3 |
| 2018 | July | D1 | 7 | 1 | 5 | 5 |
| 2018 | July | D1 | 7 | 2 | 3 | 2 |
| 2018 | July | D1 | 7 | 3 | 5 | 3 |
| 2018 | July | D1 | 8 | 1 | 5 | 2 |
| 2018 | July | D1 | 8 | 2 | 4 | 1 |
| 2018 | July | D1 | 8 | 3 | 7 | 3 |
| 2018 | July | D1 | 9 | 1 | 5 | 3 |
| 2018 | July | D1 | 9 | 2 | 5 | 1 |
| 2018 | July | D1 | 9 | 3 | 7 | 1 |
| 2018 | July | D2 | 10 | 1 | 7 | 2 |
| 2018 | July | D2 | 10 | 2 | 10 | 2 |
| 2018 | July | D2 | 10 | 3 | 10 | 2 |
| 2018 | July | D2 | 1 | 1 | 7 | 0 |
| 2018 | July | D2 | 1 | 2 | 5 | 0 |
| 2018 | July | D2 | 1 | 3 | 12 | 0 |
| 2018 | July | D2 | 2 | 1 | 6 | 5 |
| 2018 | July | D2 | 2 | 2 | 7 | 6 |
| 2018 | July | D2 | 2 | 3 | 10 | 7 |
| 2018 | July | D2 | 3 | 1 | 10 | 0 |
| 2018 | July | D2 | 3 | 2 | 5 | 0 |
| 2018 | July | D2 | 3 | 3 | 7 | 0 |
| 2018 | July | D2 | 4 | 1 | 7 | 1 |
| 2018 | July | D2 | 4 | 2 | 12 | 1 |
| 2018 | July | D2 | 4 | 3 | 2 | 0 |
| 2018 | July | D2 | 5 | 1 | 10 | 3 |
| 2018 | July | D2 | 5 | 2 | 8 | 2 |
| 2018 | July | D2 | 5 | 3 | 3 | 0 |
| 2018 | July | D2 | 6 | 1 | 8 | 0 |
| 2018 | July | D2 | 6 | 2 | 8 | 1 |
| 2018 | July | D2 | 6 | 3 | 8 | 1 |
| 2018 | July | D2 | 7 | 1 | 9 | 7 |
| 2018 | July | D2 | 7 | 2 | 7 | 5 |
| 2018 | July | D2 | 7 | 3 | 3 | 1 |
| 2018 | July | D2 | 8 | 1 | 9 | 7 |
| 2018 | July | D2 | 8 | 2 | 2 | 1 |
| 2018 | July | D2 | 8 | 3 | 10 | 5 |
| 2018 | July | D2 | 9 | 1 | 7 | 0 |
| 2018 | July | D2 | 9 | 2 | 6 | 1 |
| 2018 | July | D2 | 9 | 3 | 7 | 1 |
| 2018 | July | D3 | 10 | 1 | 6 | 2 |
| 2018 | July | D3 | 10 | 2 | 3 | 0 |
| 2018 | July | D3 | 10 | 3 | 3 | 1 |
| 2018 | July | D3 | 1 | 1 | 3 | 1 |
| 2018 | July | D3 | 1 | 2 | 6 | 2 |
| 2018 | July | D3 | 1 | 3 | 7 | 1 |
| 2018 | July | D3 | 2 | 1 | 6 | 5 |
| 2018 | July | D3 | 2 | 2 | 6 | 2 |
| 2018 | July | D3 | 2 | 3 | 7 | 2 |
| 2018 | July | D3 | 3 | 1 | 6 | 3 |
| 2018 | July | D3 | 3 | 2 | 4 | 2 |
| 2018 | July | D3 | 3 | 3 | 5 | 3 |
| 2018 | July | D3 | 4 | 1 | 8 | 2 |
| 2018 | July | D3 | 4 | 2 | 8 | 2 |
| 2018 | July | D3 | 4 | 3 | 6 | 2 |
| 2018 | July | D3 | 5 | 1 | 7 | 5 |
| 2018 | July | D3 | 5 | 2 | 6 | 4 |
| 2018 | July | D3 | 5 | 3 | 4 | 1 |
| 2018 | July | D3 | 6 | 3 | 4 | 1 |
| 2018 | July | D3 | 7 | 1 | 8 | 1 |
| 2018 | July | D3 | 7 | 2 | 8 | 4 |
| 2018 | July | D3 | 7 | 3 | 5 | 2 |
| 2018 | July | D3 | 8 | 1 | 6 | 1 |
| 2018 | July | D3 | 8 | 2 | 7 | 0 |
| 2018 | July | D3 | 8 | 3 | 6 | 1 |
| 2018 | July | D3 | 9 | 3 | 6 | 1 |
| 2018 | July | D4 | 10 | 1 | 5 | 1 |
| 2018 | July | D4 | 10 | 2 | 2 | 1 |
| 2018 | July | D4 | 10 | 3 | 6 | 1 |
| 2018 | July | D4 | 1 | 1 | 4 | 1 |
| 2018 | July | D4 | 1 | 2 | 3 | 0 |
| 2018 | July | D4 | 2 | 1 | 4 | 1 |
| 2018 | July | D4 | 2 | 3 | 6 | 1 |
| 2018 | July | D4 | 3 | 1 | 5 | 1 |
| 2018 | July | D4 | 3 | 2 | 8 | 2 |
| 2018 | July | D4 | 3 | 3 | 5 | 1 |
| 2018 | July | D4 | 4 | 1 | 6 | 2 |
| 2018 | July | D4 | 4 | 2 | 9 | 1 |
| 2018 | July | D4 | 5 | 2 | 8 | 0 |
| 2018 | July | D4 | 6 | 2 | 5 | 1 |
| 2018 | July | D4 | 6 | 3 | 8 | 1 |
| 2018 | July | D4 | 7 | 1 | 8 | 1 |
| 2018 | July | D4 | 7 | 2 | 7 | 1 |
| 2018 | July | D4 | 7 | 3 | 6 | 1 |
| 2018 | July | D4 | 8 | 1 | 7 | 1 |
| 2018 | July | D4 | 8 | 2 | 6 | 1 |
| 2018 | July | D4 | 8 | 3 | 6 | 1 |
| 2018 | July | D4 | 9 | 1 | 8 | 1 |
| 2018 | July | D4 | 9 | 2 | 8 | 1 |
| 2018 | July | D4 | 9 | 3 | 2 | 1 |
| 2018 | late August | D1 | 10 | 1 | 4 | 4 |
| 2018 | late August | D1 | 10 | 2 | 7 | 7 |
| 2018 | late August | D1 | 10 | 3 | 7 | 6 |
| 2018 | late August | D1 | 1 | 1 | 7 | 5 |
| 2018 | late August | D1 | 1 | 2 | 7 | 5 |
| 2018 | late August | D1 | 1 | 3 | 6 | 3 |
| 2018 | late August | D1 | 2 | 1 | 6 | 4 |
| 2018 | late August | D1 | 2 | 2 | 4 | 3 |
| 2018 | late August | D1 | 2 | 3 | 6 | 5 |
| 2018 | late August | D1 | 3 | 1 | 3 | 3 |
| 2018 | late August | D1 | 3 | 2 | 9 | 8 |
| 2018 | late August | D1 | 3 | 3 | 6 | 4 |
| 2018 | late August | D1 | 4 | 2 | 7 | 2 |
| 2018 | late August | D1 | 4 | 3 | 8 | 2 |
| 2018 | late August | D1 | 6 | 1 | 4 | 4 |
| 2018 | late August | D1 | 6 | 2 | 5 | 4 |
| 2018 | late August | D1 | 6 | 3 | 6 | 5 |
| 2018 | late August | D1 | 7 | 1 | 8 | 6 |
| 2018 | late August | D1 | 7 | 2 | 5 | 4 |
| 2018 | late August | D1 | 7 | 3 | 4 | 4 |
| 2018 | late August | D1 | 8 | 1 | 8 | 6 |
| 2018 | late August | D1 | 8 | 2 | 6 | 6 |
| 2018 | late August | D1 | 8 | 3 | 9 | 7 |
| 2018 | late August | D1 | 9 | 1 | 9 | 2 |
| 2018 | late August | D1 | 9 | 2 | 7 | 3 |
| 2018 | late August | D1 | 9 | 3 | 6 | 1 |
| 2018 | late August | D2 | 10 | 1 | 5 | 5 |
| 2018 | late August | D2 | 10 | 2 | 5 | 5 |
| 2018 | late August | D2 | 10 | 3 | 6 | 5 |
| 2018 | late August | D2 | 1 | 1 | 7 | 1 |
| 2018 | late August | D2 | 1 | 2 | 4 | 1 |
| 2018 | late August | D2 | 1 | 3 | 7 | 1 |
| 2018 | late August | D2 | 2 | 1 | 7 | 6 |
| 2018 | late August | D2 | 2 | 2 | 3 | 2 |
| 2018 | late August | D2 | 2 | 3 | 7 | 6 |
| 2018 | late August | D2 | 3 | 1 | 6 | 1 |
| 2018 | late August | D2 | 3 | 2 | 6 | 1 |
| 2018 | late August | D2 | 3 | 3 | 7 | 2 |
| 2018 | late August | D2 | 4 | 1 | 6 | 6 |
| 2018 | late August | D2 | 4 | 2 | 3 | 2 |
| 2018 | late August | D2 | 4 | 3 | 7 | 7 |
| 2018 | late August | D2 | 5 | 1 | 5 | 5 |
| 2018 | late August | D2 | 5 | 2 | 9 | 8 |
| 2018 | late August | D2 | 5 | 3 | 8 | 8 |
| 2018 | late August | D2 | 6 | 1 | 6 | 6 |
| 2018 | late August | D2 | 6 | 2 | 4 | 3 |
| 2018 | late August | D2 | 6 | 3 | 5 | 5 |
| 2018 | late August | D2 | 7 | 1 | 5 | 4 |
| 2018 | late August | D2 | 7 | 2 | 5 | 5 |
| 2018 | late August | D2 | 7 | 3 | 4 | 4 |
| 2018 | late August | D2 | 8 | 1 | 6 | 4 |
| 2018 | late August | D2 | 8 | 2 | 6 | 5 |
| 2018 | late August | D2 | 8 | 3 | 8 | 6 |
| 2018 | late August | D2 | 9 | 1 | 6 | 6 |
| 2018 | late August | D2 | 9 | 2 | 5 | 5 |
| 2018 | late August | D2 | 9 | 3 | 6 | 6 |
| 2018 | late August | D3 | 10 | 1 | 6 | 5 |
| 2018 | late August | D3 | 10 | 3 | 6 | 5 |
| 2018 | late August | D3 | 1 | 1 | 2 | 2 |
| 2018 | late August | D3 | 1 | 2 | 6 | 6 |
| 2018 | late August | D3 | 1 | 3 | 5 | 5 |
| 2018 | late August | D3 | 2 | 2 | 7 | 6 |
| 2018 | late August | D3 | 2 | 3 | 3 | 3 |
| 2018 | late August | D3 | 3 | 1 | 3 | 3 |
| 2018 | late August | D3 | 3 | 2 | 6 | 5 |
| 2018 | late August | D3 | 3 | 3 | 6 | 6 |
| 2018 | late August | D3 | 4 | 1 | 5 | 4 |
| 2018 | late August | D3 | 4 | 2 | 6 | 6 |
| 2018 | late August | D3 | 4 | 3 | 5 | 5 |
| 2018 | late August | D3 | 5 | 1 | 7 | 6 |
| 2018 | late August | D3 | 5 | 2 | 7 | 7 |
| 2018 | late August | D3 | 5 | 3 | 5 | 5 |
| 2018 | late August | D3 | 6 | 1 | 3 | 2 |
| 2018 | late August | D3 | 6 | 2 | 6 | 6 |
| 2018 | late August | D3 | 6 | 3 | 7 | 6 |
| 2018 | late August | D3 | 7 | 1 | 8 | 4 |
| 2018 | late August | D3 | 7 | 2 | 6 | 5 |
| 2018 | late August | D3 | 7 | 3 | 6 | 6 |
| 2018 | late August | D3 | 8 | 1 | 5 | 3 |
| 2018 | late August | D3 | 8 | 2 | 5 | 4 |
| 2018 | late August | D3 | 8 | 3 | 4 | 4 |
| 2018 | late August | D3 | 9 | 3 | 4 | 4 |
| 2018 | late August | D4 | 10 | 1 | 3 | 3 |
| 2018 | late August | D4 | 10 | 2 | 2 | 1 |
| 2018 | late August | D4 | 10 | 3 | 2 | 1 |
| 2018 | late August | D4 | 1 | 1 | 5 | 2 |
| 2018 | late August | D4 | 1 | 2 | 5 | 4 |
| 2018 | late August | D4 | 1 | 3 | 7 | 5 |
| 2018 | late August | D4 | 2 | 1 | 3 | 1 |
| 2018 | late August | D4 | 2 | 3 | 4 | 3 |
| 2018 | late August | D4 | 3 | 1 | 4 | 4 |
| 2018 | late August | D4 | 3 | 2 | 5 | 5 |
| 2018 | late August | D4 | 3 | 3 | 4 | 4 |
| 2018 | late August | D4 | 4 | 1 | 5 | 3 |
| 2018 | late August | D4 | 4 | 2 | 7 | 5 |
| 2018 | late August | D4 | 4 | 3 | 4 | 4 |
| 2018 | late August | D4 | 5 | 1 | 7 | 4 |
| 2018 | late August | D4 | 5 | 2 | 4 | 2 |
| 2018 | late August | D4 | 5 | 3 | 7 | 2 |
| 2018 | late August | D4 | 6 | 1 | 6 | 2 |
| 2018 | late August | D4 | 6 | 2 | 8 | 4 |
| 2018 | late August | D4 | 6 | 3 | 4 | 3 |
| 2018 | late August | D4 | 7 | 1 | 4 | 3 |
| 2018 | late August | D4 | 7 | 2 | 5 | 4 |
| 2018 | late August | D4 | 7 | 3 | 4 | 3 |
| 2018 | late August | D4 | 8 | 1 | 4 | 3 |
| 2018 | late August | D4 | 8 | 2 | 4 | 3 |
| 2018 | late August | D4 | 8 | 3 | 4 | 2 |
| 2018 | late August | D4 | 9 | 1 | 4 | 2 |
| 2018 | late August | D4 | 9 | 2 | 4 | 3 |
| 2018 | late August | D4 | 9 | 3 | 3 | 3 |
| 2018 | September | D1 | 10 | 1 | 5 | 5 |
| 2018 | September | D1 | 10 | 2 | 5 | 5 |
| 2018 | September | D1 | 10 | 3 | 4 | 4 |
| 2018 | September | D1 | 1 | 1 | 3 | 3 |
| 2018 | September | D1 | 1 | 2 | 6 | 6 |
| 2018 | September | D1 | 1 | 3 | 2 | 2 |
| 2018 | September | D1 | 2 | 1 | 4 | 4 |
| 2018 | September | D1 | 2 | 2 | 4 | 4 |
| 2018 | September | D1 | 2 | 3 | 2 | 2 |
| 2018 | September | D1 | 3 | 1 | 3 | 2 |
| 2018 | September | D1 | 3 | 2 | 4 | 4 |
| 2018 | September | D1 | 3 | 3 | 8 | 8 |
| 2018 | September | D1 | 4 | 1 | 4 | 1 |
| 2018 | September | D1 | 4 | 2 | 4 | 3 |
| 2018 | September | D1 | 4 | 3 | 3 | 2 |
| 2018 | September | D1 | 5 | 1 | 5 | 2 |
| 2018 | September | D1 | 5 | 2 | 5 | 1 |
| 2018 | September | D1 | 5 | 3 | 5 | 2 |
| 2018 | September | D1 | 6 | 1 | 4 | 4 |
| 2018 | September | D1 | 6 | 2 | 3 | 3 |
| 2018 | September | D1 | 6 | 3 | 3 | 3 |
| 2018 | September | D1 | 7 | 1 | 3 | 3 |
| 2018 | September | D1 | 7 | 2 | 6 | 6 |
| 2018 | September | D1 | 7 | 3 | 4 | 4 |
| 2018 | September | D1 | 8 | 1 | 4 | 3 |
| 2018 | September | D1 | 8 | 3 | 5 | 5 |
| 2018 | September | D1 | 9 | 1 | 5 | 5 |
| 2018 | September | D1 | 9 | 2 | 5 | 5 |
| 2018 | September | D1 | 9 | 3 | 4 | 3 |
| 2018 | September | D2 | 10 | 1 | 8 | 8 |
| 2018 | September | D2 | 10 | 2 | 5 | 5 |
| 2018 | September | D2 | 10 | 3 | 3 | 3 |
| 2018 | September | D2 | 1 | 1 | 5 | 1 |
| 2018 | September | D2 | 1 | 2 | 3 | 1 |
| 2018 | September | D2 | 1 | 3 | 6 | 2 |
| 2018 | September | D2 | 2 | 1 | 4 | 4 |
| 2018 | September | D2 | 2 | 2 | 3 | 3 |
| 2018 | September | D2 | 2 | 3 | 3 | 3 |
| 2018 | September | D2 | 3 | 1 | 4 | 2 |
| 2018 | September | D2 | 3 | 2 | 4 | 1 |
| 2018 | September | D2 | 3 | 3 | 4 | 2 |
| 2018 | September | D2 | 4 | 1 | 3 | 3 |
| 2018 | September | D2 | 4 | 2 | 3 | 3 |
| 2018 | September | D2 | 4 | 3 | 4 | 4 |
| 2018 | September | D2 | 5 | 1 | 6 | 6 |
| 2018 | September | D2 | 5 | 2 | 6 | 6 |
| 2018 | September | D2 | 5 | 3 | 2 | 2 |
| 2018 | September | D2 | 6 | 1 | 4 | 4 |
| 2018 | September | D2 | 6 | 2 | 3 | 3 |
| 2018 | September | D2 | 6 | 3 | 2 | 2 |
| 2018 | September | D2 | 7 | 1 | 6 | 6 |
| 2018 | September | D2 | 7 | 2 | 5 | 5 |
| 2018 | September | D2 | 7 | 3 | 3 | 3 |
| 2018 | September | D2 | 8 | 1 | 2 | 2 |
| 2018 | September | D2 | 8 | 2 | 2 | 2 |
| 2018 | September | D2 | 8 | 3 | 4 | 4 |
| 2018 | September | D2 | 9 | 1 | 4 | 4 |
| 2018 | September | D2 | 9 | 2 | 5 | 5 |
| 2018 | September | D2 | 9 | 3 | 4 | 4 |
| 2018 | September | D3 | 10 | 1 | 3 | 3 |
| 2018 | September | D3 | 1 | 1 | 3 | 3 |
| 2018 | September | D3 | 1 | 2 | 5 | 5 |
| 2018 | September | D3 | 1 | 3 | 3 | 3 |
| 2018 | September | D3 | 2 | 2 | 4 | 4 |
| 2018 | September | D3 | 2 | 3 | 3 | 3 |
| 2018 | September | D3 | 3 | 1 | 2 | 2 |
| 2018 | September | D3 | 3 | 3 | 3 | 3 |
| 2018 | September | D3 | 4 | 1 | 5 | 5 |
| 2018 | September | D3 | 4 | 2 | 4 | 3 |
| 2018 | September | D3 | 4 | 3 | 2 | 2 |
| 2018 | September | D3 | 5 | 1 | 5 | 5 |
| 2018 | September | D3 | 5 | 2 | 5 | 5 |
| 2018 | September | D3 | 5 | 3 | 5 | 5 |
| 2018 | September | D3 | 6 | 1 | 4 | 4 |
| 2018 | September | D3 | 6 | 2 | 3 | 3 |
| 2018 | September | D3 | 7 | 1 | 4 | 4 |
| 2018 | September | D3 | 7 | 3 | 4 | 4 |
| 2018 | September | D3 | 8 | 1 | 4 | 4 |
| 2018 | September | D3 | 8 | 2 | 3 | 3 |
| 2018 | September | D3 | 8 | 3 | 6 | 6 |
| 2018 | September | D3 | 9 | 3 | 3 | 3 |
| 2018 | September | D4 | 10 | 1 | 3 | 3 |
| 2018 | September | D4 | 10 | 2 | 4 | 3 |
| 2018 | September | D4 | 1 | 1 | 2 | 2 |
| 2018 | September | D4 | 1 | 2 | 3 | 3 |
| 2018 | September | D4 | 1 | 3 | 6 | 6 |
| 2018 | September | D4 | 2 | 1 | 4 | 4 |
| 2018 | September | D4 | 2 | 3 | 3 | 3 |
| 2018 | September | D4 | 3 | 1 | 5 | 5 |
| 2018 | September | D4 | 3 | 3 | 3 | 3 |
| 2018 | September | D4 | 4 | 1 | 4 | 4 |
| 2018 | September | D4 | 4 | 2 | 3 | 3 |
| 2018 | September | D4 | 4 | 3 | 4 | 4 |
| 2018 | September | D4 | 5 | 1 | 3 | 3 |
| 2018 | September | D4 | 5 | 2 | 3 | 3 |
| 2018 | September | D4 | 5 | 3 | 5 | 5 |
| 2018 | September | D4 | 6 | 1 | 7 | 7 |
| 2018 | September | D4 | 6 | 2 | 5 | 5 |
| 2018 | September | D4 | 6 | 3 | 4 | 4 |
| 2018 | September | D4 | 7 | 1 | 2 | 2 |
| 2018 | September | D4 | 7 | 2 | 2 | 2 |
| 2018 | September | D4 | 7 | 3 | 3 | 3 |
| 2018 | September | D4 | 8 | 1 | 4 | 3 |
| 2018 | September | D4 | 8 | 2 | 4 | 4 |
| 2018 | September | D4 | 8 | 3 | 3 | 3 |
| 2018 | September | D4 | 9 | 1 | 3 | 3 |
| 2018 | September | D4 | 9 | 2 | 4 | 4 |
| 2018 | September | D4 | 9 | 3 | 4 | 4 |
| 2019 | June | D1 | 1 | 1 | 6 | 0 |
| 2019 | June | D1 | 1 | 2 | 4 | 0 |
| 2019 | June | D1 | 1 | 3 | 6 | 0 |
| 2019 | June | D1 | 2 | 1 | 7 | 0 |
| 2019 | June | D1 | 2 | 2 | 4 | 0 |
| 2019 | June | D1 | 2 | 3 | 5 | 0 |
| 2019 | June | D1 | 3 | 1 | 6 | 0 |
| 2019 | June | D1 | 3 | 2 | 5 | 0 |
| 2019 | June | D1 | 3 | 3 | 4 | 0 |
| 2019 | June | D1 | 4 | 1 | 2 | 0 |
| 2019 | June | D1 | 4 | 2 | 5 | 0 |
| 2019 | June | D1 | 4 | 3 | 4 | 0 |
| 2019 | June | D1 | 5 | 1 | 2 | 0 |
| 2019 | June | D1 | 5 | 2 | 6 | 0 |
| 2019 | June | D1 | 6 | 1 | 7 | 0 |
| 2019 | June | D1 | 6 | 2 | 2 | 1 |
| 2019 | June | D1 | 6 | 3 | 3 | 0 |
| 2019 | June | D1 | 7 | 1 | 5 | 1 |
| 2019 | June | D1 | 7 | 2 | 4 | 0 |
| 2019 | June | D1 | 7 | 3 | 4 | 0 |
| 2019 | June | D1 | 8 | 1 | 5 | 0 |
| 2019 | June | D1 | 8 | 2 | 6 | 0 |
| 2019 | June | D1 | 8 | 3 | 5 | 0 |
| 2019 | June | D1 | 9 | 1 | 4 | 0 |
| 2019 | June | D1 | 9 | 2 | 5 | 1 |
| 2019 | June | D1 | 9 | 3 | 5 | 0 |
| 2019 | June | D1 | 10 | 1 | 6 | 1 |
| 2019 | June | D1 | 10 | 2 | 6 | 0 |
| 2019 | June | D1 | 10 | 3 | 5 | 0 |
| 2019 | June | D2 | 1 | 1 | 6 | 0 |
| 2019 | June | D2 | 1 | 2 | 7 | 0 |
| 2019 | June | D2 | 1 | 3 | 6 | 0 |
| 2019 | June | D2 | 2 | 1 | 4 | 0 |
| 2019 | June | D2 | 2 | 2 | 4 | 0 |
| 2019 | June | D2 | 2 | 3 | 5 | 0 |
| 2019 | June | D2 | 3 | 1 | 5 | 0 |
| 2019 | June | D2 | 3 | 2 | 6 | 0 |
| 2019 | June | D2 | 3 | 3 | 7 | 0 |
| 2019 | June | D2 | 4 | 1 | 6 | 0 |
| 2019 | June | D2 | 4 | 2 | 4 | 0 |
| 2019 | June | D2 | 4 | 3 | 6 | 0 |
| 2019 | June | D2 | 5 | 1 | 4 | 0 |
| 2019 | June | D2 | 5 | 2 | 5 | 0 |
| 2019 | June | D2 | 5 | 3 | 4 | 0 |
| 2019 | June | D2 | 6 | 1 | 7 | 0 |
| 2019 | June | D2 | 6 | 2 | 6 | 0 |
| 2019 | June | D2 | 6 | 3 | 6 | 0 |
| 2019 | June | D2 | 7 | 1 | 5 | 1 |
| 2019 | June | D2 | 7 | 2 | 6 | 0 |
| 2019 | June | D2 | 7 | 3 | 5 | 0 |
| 2019 | June | D2 | 8 | 1 | 6 | 1 |
| 2019 | June | D2 | 8 | 2 | 5 | 1 |
| 2019 | June | D2 | 8 | 3 | 5 | 1 |
| 2019 | June | D2 | 9 | 1 | 6 | 0 |
| 2019 | June | D2 | 9 | 2 | 6 | 1 |
| 2019 | June | D2 | 9 | 3 | 5 | 0 |
| 2019 | June | D2 | 10 | 1 | 7 | 0 |
| 2019 | June | D2 | 10 | 2 | 8 | 0 |
| 2019 | June | D2 | 10 | 3 | 6 | 0 |
| 2019 | June | D3 | 1 | 1 | 6 | 2 |
| 2019 | June | D3 | 1 | 2 | 7 | 2 |
| 2019 | June | D3 | 1 | 3 | 7 | 3 |
| 2019 | June | D3 | 2 | 1 | 3 | 1 |
| 2019 | June | D3 | 2 | 2 | 6 | 1 |
| 2019 | June | D3 | 2 | 3 | 2 | 1 |
| 2019 | June | D3 | 3 | 1 | 6 | 0 |
| 2019 | June | D3 | 3 | 2 | 5 | 1 |
| 2019 | June | D3 | 3 | 3 | 6 | 0 |
| 2019 | June | D3 | 4 | 1 | 6 | 0 |
| 2019 | June | D3 | 4 | 2 | 2 | 0 |
| 2019 | June | D3 | 4 | 3 | 7 | 1 |
| 2019 | June | D3 | 5 | 1 | 6 | 2 |
| 2019 | June | D3 | 5 | 2 | 3 | 1 |
| 2019 | June | D3 | 5 | 3 | 6 | 3 |
| 2019 | June | D3 | 6 | 1 | 5 | 0 |
| 2019 | June | D3 | 6 | 2 | 7 | 1 |
| 2019 | June | D3 | 6 | 3 | 3 | 1 |
| 2019 | June | D3 | 7 | 1 | 6 | 2 |
| 2019 | June | D3 | 7 | 2 | 6 | 5 |
| 2019 | June | D3 | 7 | 3 | 4 | 3 |
| 2019 | June | D3 | 8 | 1 | 7 | 1 |
| 2019 | June | D3 | 8 | 2 | 4 | 0 |
| 2019 | June | D3 | 8 | 3 | 5 | 0 |
| 2019 | June | D3 | 9 | 1 | 5 | 2 |
| 2019 | June | D3 | 9 | 2 | 7 | 3 |
| 2019 | June | D3 | 9 | 3 | 6 | 3 |
| 2019 | June | D3 | 10 | 1 | 5 | 3 |
| 2019 | June | D3 | 10 | 2 | 5 | 3 |
| 2019 | June | D3 | 10 | 3 | 6 | 3 |
| 2019 | June | D4 | 1 | 1 | 2 | 1 |
| 2019 | June | D4 | 1 | 2 | 5 | 2 |
| 2019 | June | D4 | 2 | 1 | 7 | 0 |
| 2019 | June | D4 | 2 | 2 | 4 | 1 |
| 2019 | June | D4 | 2 | 3 | 6 | 0 |
| 2019 | June | D4 | 3 | 1 | 5 | 1 |
| 2019 | June | D4 | 3 | 2 | 6 | 0 |
| 2019 | June | D4 | 3 | 3 | 6 | 0 |
| 2019 | June | D4 | 4 | 1 | 6 | 0 |
| 2019 | June | D4 | 4 | 2 | 6 | 0 |
| 2019 | June | D4 | 4 | 3 | 6 | 0 |
| 2019 | June | D4 | 5 | 1 | 4 | 0 |
| 2019 | June | D4 | 5 | 2 | 5 | 0 |
| 2019 | June | D4 | 5 | 3 | 6 | 0 |
| 2019 | June | D4 | 6 | 1 | 5 | 0 |
| 2019 | June | D4 | 6 | 2 | 4 | 0 |
| 2019 | June | D4 | 7 | 1 | 6 | 1 |
| 2019 | June | D4 | 7 | 2 | 7 | 1 |
| 2019 | June | D4 | 7 | 3 | 6 | 1 |
| 2019 | June | D4 | 8 | 1 | 5 | 3 |
| 2019 | June | D4 | 8 | 2 | 4 | 0 |
| 2019 | June | D4 | 8 | 3 | 5 | 3 |
| 2019 | June | D4 | 9 | 1 | 5 | 2 |
| 2019 | June | D4 | 9 | 2 | 5 | 1 |
| 2019 | June | D4 | 9 | 3 | 7 | 0 |
| 2019 | June | D4 | 10 | 1 | 5 | 0 |
| 2019 | June | D4 | 10 | 2 | 5 | 0 |
| 2019 | June | D4 | 10 | 3 | 2 | 0 |
| 2019 | July | D1 | 1 | 1 | 7 | 0 |
| 2019 | July | D1 | 1 | 2 | 6 | 0 |
| 2019 | July | D1 | 1 | 3 | 7 | 0 |
| 2019 | July | D1 | 2 | 1 | 5 | 1 |
| 2019 | July | D1 | 2 | 2 | 7 | 1 |
| 2019 | July | D1 | 2 | 3 | 4 | 1 |
| 2019 | July | D1 | 3 | 1 | 8 | 1 |
| 2019 | July | D1 | 3 | 2 | 8 | 4 |
| 2019 | July | D1 | 3 | 3 | 8 | 4 |
| 2019 | July | D1 | 4 | 1 | 7 | 0 |
| 2019 | July | D1 | 4 | 2 | 8 | 0 |
| 2019 | July | D1 | 4 | 3 | 9 | 0 |
| 2019 | July | D1 | 5 | 1 | 6 | 0 |
| 2019 | July | D1 | 5 | 2 | 6 | 0 |
| 2019 | July | D1 | 5 | 3 | 3 | 0 |
| 2019 | July | D1 | 6 | 1 | 7 | 0 |
| 2019 | July | D1 | 6 | 2 | 5 | 0 |
| 2019 | July | D1 | 6 | 3 | 6 | 0 |
| 2019 | July | D1 | 7 | 1 | 7 | 1 |
| 2019 | July | D1 | 7 | 2 | 8 | 1 |
| 2019 | July | D1 | 7 | 3 | 7 | 1 |
| 2019 | July | D1 | 8 | 1 | 6 | 1 |
| 2019 | July | D1 | 8 | 2 | 8 | 1 |
| 2019 | July | D1 | 8 | 3 | 5 | 1 |
| 2019 | July | D1 | 9 | 1 | 5 | 1 |
| 2019 | July | D1 | 9 | 2 | 6 | 0 |
| 2019 | July | D1 | 9 | 3 | 4 | 0 |
| 2019 | July | D1 | 10 | 1 | 5 | 1 |
| 2019 | July | D1 | 10 | 2 | 6 | 0 |
| 2019 | July | D1 | 10 | 3 | 8 | 2 |
| 2019 | July | D2 | 1 | 1 | 3 | 0 |
| 2019 | July | D2 | 1 | 2 | 7 | 0 |
| 2019 | July | D2 | 1 | 3 | 7 | 0 |
| 2019 | July | D2 | 2 | 1 | 7 | 6 |
| 2019 | July | D2 | 2 | 2 | 6 | 5 |
| 2019 | July | D2 | 2 | 3 | 8 | 6 |
| 2019 | July | D2 | 3 | 1 | 8 | 0 |
| 2019 | July | D2 | 3 | 2 | 7 | 0 |
| 2019 | July | D2 | 3 | 3 | 8 | 0 |
| 2019 | July | D2 | 4 | 1 | 9 | 2 |
| 2019 | July | D2 | 4 | 2 | 10 | 1 |
| 2019 | July | D2 | 4 | 3 | 9 | 1 |
| 2019 | July | D2 | 5 | 1 | 9 | 2 |
| 2019 | July | D2 | 5 | 2 | 8 | 1 |
| 2019 | July | D2 | 5 | 3 | 9 | 2 |
| 2019 | July | D2 | 6 | 1 | 7 | 2 |
| 2019 | July | D2 | 6 | 2 | 7 | 2 |
| 2019 | July | D2 | 6 | 3 | 8 | 3 |
| 2019 | July | D2 | 7 | 1 | 7 | 3 |
| 2019 | July | D2 | 7 | 2 | 8 | 3 |
| 2019 | July | D2 | 7 | 3 | 9 | 8 |
| 2019 | July | D2 | 8 | 1 | 7 | 5 |
| 2019 | July | D2 | 8 | 2 | 7 | 5 |
| 2019 | July | D2 | 8 | 3 | 6 | 3 |
| 2019 | July | D2 | 9 | 1 | 5 | 1 |
| 2019 | July | D2 | 9 | 2 | 6 | 1 |
| 2019 | July | D2 | 9 | 3 | 5 | 3 |
| 2019 | July | D2 | 10 | 1 | 8 | 5 |
| 2019 | July | D2 | 10 | 2 | 7 | 4 |
| 2019 | July | D2 | 10 | 3 | 9 | 6 |
| 2019 | July | D3 | 1 | 1 | 9 | 3 |
| 2019 | July | D3 | 1 | 2 | 6 | 1 |
| 2019 | July | D3 | 1 | 3 | 6 | 3 |
| 2019 | July | D3 | 2 | 1 | 8 | 2 |
| 2019 | July | D3 | 2 | 2 | 7 | 4 |
| 2019 | July | D3 | 2 | 3 | 6 | 2 |
| 2019 | July | D3 | 3 | 1 | 6 | 1 |
| 2019 | July | D3 | 3 | 2 | 7 | 1 |
| 2019 | July | D3 | 3 | 3 | 7 | 1 |
| 2019 | July | D3 | 4 | 1 | 9 | 2 |
| 2019 | July | D3 | 4 | 3 | 4 | 2 |
| 2019 | July | D3 | 5 | 1 | 7 | 3 |
| 2019 | July | D3 | 5 | 2 | 6 | 3 |
| 2019 | July | D3 | 5 | 3 | 7 | 2 |
| 2019 | July | D3 | 6 | 1 | 7 | 2 |
| 2019 | July | D3 | 6 | 2 | 6 | 2 |
| 2019 | July | D3 | 6 | 3 | 5 | 1 |
| 2019 | July | D3 | 7 | 1 | 6 | 0 |
| 2019 | July | D3 | 7 | 2 | 7 | 2 |
| 2019 | July | D3 | 7 | 3 | 7 | 1 |
| 2019 | July | D3 | 8 | 1 | 7 | 1 |
| 2019 | July | D3 | 8 | 2 | 7 | 1 |
| 2019 | July | D3 | 8 | 3 | 8 | 1 |
| 2019 | July | D3 | 9 | 1 | 7 | 3 |
| 2019 | July | D3 | 9 | 2 | 7 | 2 |
| 2019 | July | D3 | 9 | 3 | 7 | 1 |
| 2019 | July | D3 | 10 | 1 | 4 | 1 |
| 2019 | July | D3 | 10 | 2 | 4 | 1 |
| 2019 | July | D3 | 10 | 3 | 7 | 1 |
| 2019 | July | D4 | 1 | 1 | 5 | 1 |
| 2019 | July | D4 | 1 | 2 | 5 | 2 |
| 2019 | July | D4 | 1 | 3 | 6 | 2 |
| 2019 | July | D4 | 2 | 1 | 7 | 2 |
| 2019 | July | D4 | 2 | 2 | 5 | 1 |
| 2019 | July | D4 | 2 | 3 | 5 | 2 |
| 2019 | July | D4 | 3 | 1 | 7 | 3 |
| 2019 | July | D4 | 3 | 2 | 7 | 3 |
| 2019 | July | D4 | 3 | 3 | 6 | 2 |
| 2019 | July | D4 | 4 | 1 | 8 | 3 |
| 2019 | July | D4 | 4 | 2 | 7 | 1 |
| 2019 | July | D4 | 4 | 3 | 8 | 1 |
| 2019 | July | D4 | 5 | 1 | 5 | 1 |
| 2019 | July | D4 | 5 | 2 | 4 | 0 |
| 2019 | July | D4 | 5 | 3 | 5 | 2 |
| 2019 | July | D4 | 6 | 1 | 5 | 0 |
| 2019 | July | D4 | 6 | 2 | 6 | 1 |
| 2019 | July | D4 | 6 | 3 | 5 | 0 |
| 2019 | July | D4 | 7 | 1 | 7 | 5 |
| 2019 | July | D4 | 7 | 2 | 5 | 2 |
| 2019 | July | D4 | 7 | 3 | 8 | 4 |
| 2019 | July | D4 | 8 | 1 | 6 | 2 |
| 2019 | July | D4 | 8 | 2 | 7 | 2 |
| 2019 | July | D4 | 8 | 3 | 6 | 3 |
| 2019 | July | D4 | 9 | 1 | 4 | 1 |
| 2019 | July | D4 | 9 | 2 | 6 | 1 |
| 2019 | July | D4 | 9 | 3 | 5 | 1 |
| 2019 | July | D4 | 10 | 1 | 8 | 4 |
| 2019 | July | D4 | 10 | 2 | 7 | 2 |
| 2019 | July | D4 | 10 | 3 | 5 | 0 |
| 2019 | early August | D1 | 1 | 1 | 6 | 1 |
| 2019 | early August | D1 | 1 | 2 | 6 | 0 |
| 2019 | early August | D1 | 1 | 3 | 6 | 1 |
| 2019 | early August | D1 | 2 | 1 | 6 | 2 |
| 2019 | early August | D1 | 2 | 2 | 2 | 1 |
| 2019 | early August | D1 | 2 | 3 | 5 | 1 |
| 2019 | early August | D1 | 3 | 1 | 2 | 1 |
| 2019 | early August | D1 | 3 | 2 | 9 | 2 |
| 2019 | early August | D1 | 3 | 3 | 7 | 2 |
| 2019 | early August | D1 | 4 | 1 | 4 | 0 |
| 2019 | early August | D1 | 4 | 2 | 5 | 1 |
| 2019 | early August | D1 | 4 | 3 | 6 | 1 |
| 2019 | early August | D1 | 5 | 1 | 5 | 0 |
| 2019 | early August | D1 | 5 | 2 | 3 | 0 |
| 2019 | early August | D1 | 6 | 1 | 7 | 1 |
| 2019 | early August | D1 | 6 | 2 | 8 | 1 |
| 2019 | early August | D1 | 6 | 3 | 5 | 2 |
| 2019 | early August | D1 | 7 | 1 | 10 | 1 |
| 2019 | early August | D1 | 7 | 2 | 7 | 3 |
| 2019 | early August | D1 | 7 | 3 | 7 | 1 |
| 2019 | early August | D1 | 8 | 1 | 5 | 1 |
| 2019 | early August | D1 | 8 | 2 | 3 | 1 |
| 2019 | early August | D1 | 8 | 3 | 6 | 2 |
| 2019 | early August | D1 | 9 | 1 | 7 | 1 |
| 2019 | early August | D1 | 9 | 2 | 6 | 1 |
| 2019 | early August | D1 | 9 | 3 | 5 | 0 |
| 2019 | early August | D1 | 10 | 1 | 3 | 1 |
| 2019 | early August | D1 | 10 | 2 | 7 | 2 |
| 2019 | early August | D1 | 10 | 3 | 5 | 1 |
| 2019 | early August | D2 | 1 | 1 | 7 | 1 |
| 2019 | early August | D2 | 1 | 2 | 4 | 0 |
| 2019 | early August | D2 | 1 | 3 | 6 | 0 |
| 2019 | early August | D2 | 2 | 1 | 6 | 5 |
| 2019 | early August | D2 | 2 | 2 | 10 | 9 |
| 2019 | early August | D2 | 2 | 3 | 7 | 6 |
| 2019 | early August | D2 | 3 | 1 | 2 | 1 |
| 2019 | early August | D2 | 3 | 2 | 3 | 1 |
| 2019 | early August | D2 | 3 | 3 | 8 | 2 |
| 2019 | early August | D2 | 4 | 1 | 5 | 2 |
| 2019 | early August | D2 | 4 | 2 | 6 | 3 |
| 2019 | early August | D2 | 4 | 3 | 6 | 3 |
| 2019 | early August | D2 | 5 | 1 | 4 | 2 |
| 2019 | early August | D2 | 5 | 2 | 7 | 2 |
| 2019 | early August | D2 | 5 | 3 | 7 | 1 |
| 2019 | early August | D2 | 6 | 1 | 7 | 4 |
| 2019 | early August | D2 | 6 | 2 | 8 | 3 |
| 2019 | early August | D2 | 6 | 3 | 7 | 3 |
| 2019 | early August | D2 | 7 | 1 | 10 | 7 |
| 2019 | early August | D2 | 7 | 2 | 9 | 5 |
| 2019 | early August | D2 | 7 | 3 | 8 | 6 |
| 2019 | early August | D2 | 8 | 1 | 8 | 6 |
| 2019 | early August | D2 | 8 | 2 | 8 | 6 |
| 2019 | early August | D2 | 8 | 3 | 9 | 8 |
| 2019 | early August | D2 | 9 | 1 | 7 | 2 |
| 2019 | early August | D2 | 9 | 2 | 6 | 1 |
| 2019 | early August | D2 | 9 | 3 | 7 | 2 |
| 2019 | early August | D2 | 10 | 1 | 6 | 4 |
| 2019 | early August | D2 | 10 | 2 | 7 | 4 |
| 2019 | early August | D2 | 10 | 3 | 7 | 5 |
| 2019 | early August | D3 | 1 | 1 | 7 | 3 |
| 2019 | early August | D3 | 1 | 2 | 9 | 3 |
| 2019 | early August | D3 | 1 | 3 | 7 | 4 |
| 2019 | early August | D3 | 2 | 1 | 6 | 3 |
| 2019 | early August | D3 | 2 | 2 | 8 | 7 |
| 2019 | early August | D3 | 2 | 3 | 3 | 1 |
| 2019 | early August | D3 | 3 | 1 | 9 | 1 |
| 2019 | early August | D3 | 3 | 2 | 2 | 1 |
| 2019 | early August | D3 | 3 | 3 | 7 | 2 |
| 2019 | early August | D3 | 4 | 1 | 7 | 4 |
| 2019 | early August | D3 | 4 | 2 | 9 | 2 |
| 2019 | early August | D3 | 4 | 3 | 6 | 2 |
| 2019 | early August | D3 | 5 | 1 | 6 | 4 |
| 2019 | early August | D3 | 5 | 2 | 5 | 3 |
| 2019 | early August | D3 | 5 | 3 | 6 | 3 |
| 2019 | early August | D3 | 6 | 1 | 6 | 4 |
| 2019 | early August | D3 | 6 | 2 | 7 | 5 |
| 2019 | early August | D3 | 6 | 3 | 8 | 5 |
| 2019 | early August | D3 | 7 | 1 | 3 | 1 |
| 2019 | early August | D3 | 7 | 2 | 2 | 1 |
| 2019 | early August | D3 | 7 | 3 | 2 | 1 |
| 2019 | early August | D3 | 8 | 1 | 5 | 1 |
| 2019 | early August | D3 | 8 | 2 | 6 | 1 |
| 2019 | early August | D3 | 8 | 3 | 5 | 1 |
| 2019 | early August | D3 | 9 | 1 | 3 | 1 |
| 2019 | early August | D3 | 9 | 2 | 5 | 1 |
| 2019 | early August | D3 | 9 | 3 | 2 | 1 |
| 2019 | early August | D3 | 10 | 1 | 3 | 1 |
| 2019 | early August | D3 | 10 | 2 | 4 | 1 |
| 2019 | early August | D3 | 10 | 3 | 2 | 1 |
| 2019 | early August | D4 | 1 | 1 | 5 | 1 |
| 2019 | early August | D4 | 1 | 2 | 5 | 1 |
| 2019 | early August | D4 | 1 | 3 | 4 | 1 |
| 2019 | early August | D4 | 2 | 1 | 5 | 2 |
| 2019 | early August | D4 | 2 | 2 | 4 | 2 |
| 2019 | early August | D4 | 2 | 3 | 5 | 4 |
| 2019 | early August | D4 | 3 | 1 | 5 | 4 |
| 2019 | early August | D4 | 3 | 2 | 5 | 4 |
| 2019 | early August | D4 | 3 | 3 | 8 | 3 |
| 2019 | early August | D4 | 4 | 1 | 6 | 5 |
| 2019 | early August | D4 | 4 | 2 | 6 | 4 |
| 2019 | early August | D4 | 4 | 3 | 6 | 4 |
| 2019 | early August | D4 | 5 | 1 | 6 | 1 |
| 2019 | early August | D4 | 5 | 2 | 2 | 1 |
| 2019 | early August | D4 | 5 | 3 | 6 | 1 |
| 2019 | early August | D4 | 6 | 1 | 5 | 1 |
| 2019 | early August | D4 | 6 | 2 | 7 | 2 |
| 2019 | early August | D4 | 6 | 3 | 6 | 1 |
| 2019 | early August | D4 | 7 | 1 | 4 | 2 |
| 2019 | early August | D4 | 7 | 2 | 8 | 4 |
| 2019 | early August | D4 | 7 | 3 | 7 | 6 |
| 2019 | early August | D4 | 8 | 1 | 5 | 3 |
| 2019 | early August | D4 | 8 | 2 | 5 | 1 |
| 2019 | early August | D4 | 8 | 3 | 6 | 1 |
| 2019 | early August | D4 | 9 | 1 | 5 | 1 |
| 2019 | early August | D4 | 9 | 2 | 5 | 1 |
| 2019 | early August | D4 | 9 | 3 | 6 | 2 |
| 2019 | early August | D4 | 10 | 1 | 5 | 3 |
| 2019 | early August | D4 | 10 | 2 | 5 | 3 |
| 2019 | early August | D4 | 10 | 3 | 5 | 3 |
| 2019 | late August | D1 | 1 | 1 | 2 | 1 |
| 2019 | late August | D1 | 1 | 2 | 7 | 2 |
| 2019 | late August | D1 | 1 | 3 | 9 | 1 |
| 2019 | late August | D1 | 2 | 1 | 5 | 1 |
| 2019 | late August | D1 | 2 | 2 | 6 | 2 |
| 2019 | late August | D1 | 2 | 3 | 9 | 1 |
| 2019 | late August | D1 | 3 | 1 | 7 | 1 |
| 2019 | late August | D1 | 3 | 2 | 6 | 2 |
| 2019 | late August | D1 | 3 | 3 | 9 | 1 |
| 2019 | late August | D1 | 4 | 1 | 8 | 0 |
| 2019 | late August | D1 | 4 | 2 | 6 | 0 |
| 2019 | late August | D1 | 4 | 3 | 7 | 0 |
| 2019 | late August | D1 | 5 | 1 | 7 | 0 |
| 2019 | late August | D1 | 5 | 2 | 5 | 0 |
| 2019 | late August | D1 | 6 | 1 | 5 | 1 |
| 2019 | late August | D1 | 6 | 2 | 7 | 1 |
| 2019 | late August | D1 | 6 | 3 | 5 | 1 |
| 2019 | late August | D1 | 7 | 1 | 6 | 1 |
| 2019 | late August | D1 | 7 | 2 | 6 | 1 |
| 2019 | late August | D1 | 7 | 3 | 5 | 1 |
| 2019 | late August | D1 | 8 | 1 | 6 | 1 |
| 2019 | late August | D1 | 8 | 2 | 6 | 1 |
| 2019 | late August | D1 | 8 | 3 | 7 | 2 |
| 2019 | late August | D1 | 9 | 1 | 7 | 1 |
| 2019 | late August | D1 | 9 | 2 | 8 | 1 |
| 2019 | late August | D1 | 9 | 3 | 4 | 1 |
| 2019 | late August | D1 | 10 | 1 | 5 | 1 |
| 2019 | late August | D1 | 10 | 2 | 5 | 2 |
| 2019 | late August | D1 | 10 | 3 | 5 | 1 |
| 2019 | late August | D2 | 1 | 1 | 6 | 2 |
| 2019 | late August | D2 | 1 | 2 | 7 | 1 |
| 2019 | late August | D2 | 1 | 3 | 6 | 0 |
| 2019 | late August | D2 | 2 | 1 | 5 | 2 |
| 2019 | late August | D2 | 2 | 2 | 7 | 4 |
| 2019 | late August | D2 | 2 | 3 | 6 | 3 |
| 2019 | late August | D2 | 3 | 1 | 7 | 1 |
| 2019 | late August | D2 | 3 | 2 | 9 | 1 |
| 2019 | late August | D2 | 3 | 3 | 5 | 1 |
| 2019 | late August | D2 | 4 | 1 | 7 | 2 |
| 2019 | late August | D2 | 4 | 2 | 6 | 2 |
| 2019 | late August | D2 | 4 | 3 | 6 | 1 |
| 2019 | late August | D2 | 5 | 1 | 2 | 1 |
| 2019 | late August | D2 | 5 | 2 | 6 | 1 |
| 2019 | late August | D2 | 5 | 3 | 5 | 1 |
| 2019 | late August | D2 | 6 | 1 | 5 | 2 |
| 2019 | late August | D2 | 6 | 2 | 6 | 3 |
| 2019 | late August | D2 | 6 | 3 | 7 | 1 |
| 2019 | late August | D2 | 7 | 1 | 6 | 2 |
| 2019 | late August | D2 | 7 | 2 | 5 | 2 |
| 2019 | late August | D2 | 7 | 3 | 5 | 1 |
| 2019 | late August | D2 | 8 | 1 | 2 | 1 |
| 2019 | late August | D2 | 8 | 2 | 7 | 5 |
| 2019 | late August | D2 | 8 | 3 | 7 | 6 |
| 2019 | late August | D2 | 9 | 1 | 6 | 2 |
| 2019 | late August | D2 | 9 | 2 | 7 | 1 |
| 2019 | late August | D2 | 9 | 3 | 7 | 3 |
| 2019 | late August | D2 | 10 | 1 | 6 | 3 |
| 2019 | late August | D2 | 10 | 2 | 6 | 2 |
| 2019 | late August | D2 | 10 | 3 | 6 | 2 |
| 2019 | late August | D3 | 1 | 1 | 5 | 3 |
| 2019 | late August | D3 | 1 | 2 | 6 | 2 |
| 2019 | late August | D3 | 1 | 3 | 5 | 3 |
| 2019 | late August | D3 | 2 | 1 | 5 | 1 |
| 2019 | late August | D3 | 2 | 2 | 6 | 2 |
| 2019 | late August | D3 | 2 | 3 | 5 | 2 |
| 2019 | late August | D3 | 3 | 1 | 3 | 1 |
| 2019 | late August | D3 | 3 | 2 | 3 | 1 |
| 2019 | late August | D3 | 3 | 3 | 4 | 1 |
| 2019 | late August | D3 | 4 | 1 | 5 | 2 |
| 2019 | late August | D3 | 4 | 2 | 6 | 3 |
| 2019 | late August | D3 | 4 | 3 | 5 | 1 |
| 2019 | late August | D3 | 5 | 1 | 7 | 3 |
| 2019 | late August | D3 | 5 | 2 | 5 | 2 |
| 2019 | late August | D3 | 5 | 3 | 5 | 3 |
| 2019 | late August | D3 | 6 | 1 | 6 | 2 |
| 2019 | late August | D3 | 6 | 2 | 5 | 1 |
| 2019 | late August | D3 | 6 | 3 | 7 | 4 |
| 2019 | late August | D3 | 7 | 1 | 7 | 1 |
| 2019 | late August | D3 | 7 | 2 | 4 | 1 |
| 2019 | late August | D3 | 7 | 3 | 5 | 1 |
| 2019 | late August | D3 | 8 | 1 | 6 | 1 |
| 2019 | late August | D3 | 8 | 2 | 5 | 1 |
| 2019 | late August | D3 | 8 | 3 | 5 | 1 |
| 2019 | late August | D3 | 9 | 1 | 6 | 1 |
| 2019 | late August | D3 | 9 | 2 | 4 | 1 |
| 2019 | late August | D3 | 9 | 3 | 4 | 1 |
| 2019 | late August | D3 | 10 | 1 | 6 | 1 |
| 2019 | late August | D3 | 10 | 2 | 2 | 1 |
| 2019 | late August | D3 | 10 | 3 | 6 | 1 |
| 2019 | late August | D4 | 1 | 1 | 6 | 1 |
| 2019 | late August | D4 | 1 | 2 | 6 | 1 |
| 2019 | late August | D4 | 1 | 3 | 6 | 1 |
| 2019 | late August | D4 | 2 | 1 | 7 | 3 |
| 2019 | late August | D4 | 2 | 2 | 5 | 2 |
| 2019 | late August | D4 | 2 | 3 | 8 | 2 |
| 2019 | late August | D4 | 3 | 1 | 9 | 8 |
| 2019 | late August | D4 | 3 | 2 | 8 | 6 |
| 2019 | late August | D4 | 3 | 3 | 8 | 7 |
| 2019 | late August | D4 | 4 | 1 | 6 | 5 |
| 2019 | late August | D4 | 4 | 2 | 4 | 3 |
| 2019 | late August | D4 | 4 | 3 | 6 | 6 |
| 2019 | late August | D4 | 5 | 1 | 5 | 1 |
| 2019 | late August | D4 | 5 | 2 | 5 | 1 |
| 2019 | late August | D4 | 5 | 3 | 6 | 1 |
| 2019 | late August | D4 | 6 | 1 | 8 | 2 |
| 2019 | late August | D4 | 6 | 2 | 6 | 1 |
| 2019 | late August | D4 | 6 | 3 | 8 | 2 |
| 2019 | late August | D4 | 7 | 1 | 6 | 6 |
| 2019 | late August | D4 | 7 | 2 | 8 | 6 |
| 2019 | late August | D4 | 7 | 3 | 7 | 6 |
| 2019 | late August | D4 | 8 | 1 | 5 | 1 |
| 2019 | late August | D4 | 8 | 2 | 5 | 1 |
| 2019 | late August | D4 | 8 | 3 | 7 | 3 |
| 2019 | late August | D4 | 9 | 1 | 7 | 3 |
| 2019 | late August | D4 | 9 | 2 | 4 | 2 |
| 2019 | late August | D4 | 9 | 3 | 5 | 1 |
| 2019 | late August | D4 | 10 | 1 | 6 | 5 |
| 2019 | late August | D4 | 10 | 2 | 6 | 4 |
| 2019 | late August | D4 | 10 | 3 | 7 | 3 |
| 2019 | September | D2 | 1 | 1 | 2 | 0 |
| 2019 | September | D2 | 1 | 2 | 2 | 0 |
| 2019 | September | D2 | 1 | 3 | 3 | 0 |
| 2019 | September | D2 | 2 | 1 | 2 | 2 |
| 2019 | September | D2 | 2 | 2 | 3 | 1 |
| 2019 | September | D2 | 2 | 3 | 5 | 5 |
| 2019 | September | D2 | 3 | 1 | 4 | 1 |
| 2019 | September | D2 | 3 | 2 | 3 | 1 |
| 2019 | September | D2 | 3 | 3 | 3 | 2 |
| 2019 | September | D2 | 4 | 1 | 2 | 1 |
| 2019 | September | D2 | 4 | 2 | 3 | 0 |
| 2019 | September | D2 | 4 | 3 | 3 | 1 |
| 2019 | September | D2 | 5 | 1 | 4 | 2 |
| 2019 | September | D2 | 5 | 2 | 3 | 2 |
| 2019 | September | D2 | 5 | 3 | 3 | 1 |
| 2019 | September | D2 | 6 | 1 | 3 | 2 |
| 2019 | September | D2 | 6 | 2 | 4 | 1 |
| 2019 | September | D2 | 6 | 3 | 2 | 1 |
| 2019 | September | D2 | 7 | 1 | 4 | 1 |
| 2019 | September | D2 | 7 | 2 | 3 | 1 |
| 2019 | September | D2 | 7 | 3 | 5 | 5 |
| 2019 | September | D2 | 8 | 1 | 4 | 2 |
| 2019 | September | D2 | 8 | 2 | 4 | 4 |
| 2019 | September | D2 | 8 | 3 | 3 | 2 |
| 2019 | September | D2 | 9 | 1 | 3 | 2 |
| 2019 | September | D2 | 9 | 2 | 3 | 1 |
| 2019 | September | D2 | 9 | 3 | 3 | 0 |
| 2019 | September | D2 | 10 | 1 | 2 | 1 |
| 2019 | September | D2 | 10 | 2 | 2 | 1 |
| 2019 | September | D2 | 10 | 3 | 3 | 1 |
| 2019 | September | D3 | 1 | 1 | 3 | 1 |
| 2019 | September | D3 | 1 | 2 | 2 | 1 |
| 2019 | September | D3 | 1 | 3 | 2 | 2 |
| 2019 | September | D3 | 2 | 1 | 2 | 2 |
| 2019 | September | D3 | 2 | 2 | 3 | 2 |
| 2019 | September | D3 | 2 | 3 | 2 | 2 |
| 2019 | September | D3 | 3 | 1 | 2 | 1 |
| 2019 | September | D3 | 3 | 2 | 2 | 1 |
| 2019 | September | D3 | 3 | 3 | 3 | 1 |
| 2019 | September | D3 | 4 | 1 | 4 | 2 |
| 2019 | September | D3 | 4 | 2 | 3 | 2 |
| 2019 | September | D3 | 4 | 3 | 3 | 1 |
| 2019 | September | D3 | 5 | 1 | 3 | 3 |
| 2019 | September | D3 | 5 | 2 | 3 | 2 |
| 2019 | September | D3 | 5 | 3 | 2 | 2 |
| 2019 | September | D3 | 6 | 1 | 1 | 1 |
| 2019 | September | D3 | 6 | 2 | 3 | 3 |
| 2019 | September | D3 | 6 | 3 | 1 | 1 |
| 2019 | September | D3 | 7 | 1 | 3 | 1 |
| 2019 | September | D3 | 7 | 2 | 3 | 1 |
| 2019 | September | D3 | 7 | 3 | 3 | 0 |
| 2019 | September | D3 | 8 | 1 | 2 | 1 |
| 2019 | September | D3 | 8 | 2 | 1 | 1 |
| 2019 | September | D3 | 8 | 3 | 2 | 1 |
| 2019 | September | D3 | 9 | 1 | 3 | 1 |
| 2019 | September | D3 | 9 | 2 | 1 | 0 |
| 2019 | September | D3 | 9 | 3 | 1 | 0 |
| 2019 | September | D3 | 10 | 1 | 2 | 1 |
| 2019 | September | D3 | 10 | 2 | 1 | 1 |
| 2019 | September | D3 | 10 | 3 | 2 | 0 |
| 2019 | September | D4 | 1 | 1 | 5 | 3 |
| 2019 | September | D4 | 1 | 2 | 3 | 2 |
| 2019 | September | D4 | 1 | 3 | 3 | 1 |
| 2019 | September | D4 | 2 | 1 | 4 | 2 |
| 2019 | September | D4 | 2 | 2 | 4 | 4 |
| 2019 | September | D4 | 2 | 3 | 2 | 1 |
| 2019 | September | D4 | 3 | 1 | 6 | 6 |
| 2019 | September | D4 | 3 | 2 | 6 | 6 |
| 2019 | September | D4 | 3 | 3 | 5 | 5 |
| 2019 | September | D4 | 4 | 1 | 3 | 3 |
| 2019 | September | D4 | 4 | 2 | 3 | 3 |
| 2019 | September | D4 | 4 | 3 | 4 | 4 |
| 2019 | September | D4 | 5 | 1 | 3 | 1 |
| 2019 | September | D4 | 5 | 2 | 3 | 1 |
| 2019 | September | D4 | 5 | 3 | 3 | 1 |
| 2019 | September | D4 | 6 | 1 | 4 | 2 |
| 2019 | September | D4 | 6 | 2 | 5 | 2 |
| 2019 | September | D4 | 6 | 3 | 6 | 3 |
| 2019 | September | D4 | 7 | 1 | 5 | 4 |
| 2019 | September | D4 | 7 | 2 | 6 | 6 |
| 2019 | September | D4 | 7 | 3 | 6 | 6 |
| 2019 | September | D4 | 8 | 1 | 2 | 2 |
| 2019 | September | D4 | 8 | 2 | 2 | 1 |
| 2019 | September | D4 | 8 | 3 | 3 | 0 |
| 2019 | September | D4 | 9 | 1 | 3 | 0 |
| 2019 | September | D4 | 9 | 2 | 3 | 0 |
| 2019 | September | D4 | 9 | 3 | 4 | 0 |
| 2019 | September | D4 | 10 | 1 | 4 | 4 |
| 2019 | September | D4 | 10 | 2 | 4 | 4 |
| 2019 | September | D4 | 10 | 3 | 4 | 4 |
