# Supplementary material for: Emerging fungal pathogen of an invasive grass: Implications for competition with native plant species
Source: PLoS One. 2021 Mar 1;16(3):e0237894. doi: 10.1371/journal.pone.0237894 (PMC7920361; doi:10.1371/journal.pone.0237894)
Supplement: S3 Table — Raw data collected from an experiment at BONWR in which fungicide or water (control) were added to plots with ten planting treatments. Leaves of Microstegium vimineum (Mv) and Elymus virginicus (Ev) were assessed for B. gigantea conidiophores using microscopy. Bipolaris gigantea was isolated, as well as some co-occurring fungi, including Pyricularia spp., Bipolaris spp. other than B. gigantea, and Curvularia spp. Leaves were collected in late August of 2018. (DOCX) [file pone.0237894.s004.docx]

**S3 Table. Fungi identification on field-collected leaves.** Raw data collected from an experiment at BONWR in which fungicide or water (control) were added to plots with ten planting treatments. Leaves of *Microstegium vimineum* (Mv) and *Elymus virginicus* (Ev) were assessed for *B. gigantea* conidiophores using microscopy. *Bipolaris gigantea* was isolated, as well as some co-occurring fungi, including *Pyricularia* spp., *Bipolaris* spp. other than *B. gigantea*, and *Curvularia* spp. Leaves were collected in late August of 2018.

| Site | Plot | Treatment | Sp | *B. gigantea* | *B. gigantea* isolated | *Pyricularia* isolated | other *Bipolaris* isolated | *Curvularia* isolated |
| --- | --- | --- | --- | --- | --- | --- | --- | --- |
| D1 | 8 | fungicide | Mv | Yes | 1 | 0 | 0 | 0 |
| D1 | 10 | fungicide | Mv | Yes | 1 | 1 | 0 | 0 |
| D1 | 2 | water | Mv | Yes | 1 | 0 | 0 | 0 |
| D1 | 6 | water | Mv | Yes | 2 | 0 | 0 | 0 |
| D1 | 7 | water | Mv | Yes | 1 | 1 | 0 | 0 |
| D1 | 9 | water | Mv | Yes | 1 | 0 | 0 | 0 |
| D1 | 2 | fungicide | Ev | Yes | 0 | 0 | 0 | 0 |
| D1 | 3 | fungicide | Ev | No | 0 | 0 | 0 | 0 |
| D1 | 4 | fungicide | Ev | No | 0 | 0 | 0 | 0 |
| D1 | 6 | fungicide | Ev | No | 0 | 0 | 0 | 0 |
| D1 | 8 | fungicide | Ev | Yes | 0 | 0 | 0 | 0 |
| D1 | 9 | fungicide | Ev | No | 0 | 0 | 0 | 0 |
| D1 | 10 | fungicide | Ev | No | 0 | 0 | 0 | 0 |
| D1 | 1 | water | Ev | No | 0 | 0 | 0 | 0 |
| D1 | 2 | water | Ev | No | 0 | 0 | 0 | 0 |
| D1 | 3 | water | Ev | Yes | 0 | 0 | 0 | 0 |
| D1 | 6 | water | Ev | Yes | 0 | 0 | 0 | 0 |
| D1 | 7 | water | Ev | Yes | 0 | 0 | 0 | 0 |
| D1 | 9 | water | Ev | Yes | 0 | 0 | 0 | 0 |
| D1 | 10 | water | Ev | Yes | 0 | 0 | 0 | 0 |
| D2 | 1 | fungicide | Mv | Yes | 1 | 0 | 0 | 0 |
| D2 | 3 | fungicide | Mv | Yes | 1 | 0 | 0 | 0 |
| D2 | 5 | fungicide | Mv | Yes | 1 | 0 | 0 | 0 |
| D2 | 6 | fungicide | Mv | Yes | 1 | 0 | 0 | 0 |
| D2 | 8 | fungicide | Mv | Yes | 1 | 0 | 0 | 0 |
| D2 | 2 | water | Mv | Yes | 1 | 0 | 0 | 0 |
| D2 | 3 | water | Mv | Yes | 1 | 0 | 0 | 0 |
| D2 | 4 | water | Mv | Yes | 2 | 0 | 0 | 0 |
| D2 | 5 | fungicide | Ev | No | 0 | 0 | 0 | 0 |
| D2 | 6 | fungicide | Ev | No | 0 | 0 | 0 | 0 |
| D2 | 8 | fungicide | Ev | Yes | 0 | 0 | 0 | 0 |
| D2 | 3 | water | Ev | No | 0 | 0 | 0 | 0 |
| D2 | 6 | water | Ev | Yes | 0 | 0 | 0 | 0 |
| D2 | 7 | water | Ev | Yes | 0 | 0 | 0 | 0 |
| D2 | 8 | water | Ev | Yes | 0 | 0 | 0 | 0 |
| D2 | 9 | water | Ev | No | 0 | 0 | 0 | 0 |
| D2 | 10 | water | Ev | Yes | 0 | 0 | 0 | 0 |
| D3 | 1 | fungicide | Mv | Yes | 1 | 0 | 0 | 0 |
| D3 | 4 | fungicide | Mv | Yes | 1 | 0 | 1 | 0 |
| D3 | 6 | fungicide | Mv | Yes | 2 | 0 | 0 | 0 |
| D3 | 9 | fungicide | Mv | Yes | 1 | 0 | 0 | 0 |
| D3 | 2 | water | Mv | Yes | 1 | 0 | 0 | 0 |
| D3 | 3 | water | Mv | Yes | 1 | 0 | 0 | 0 |
| D3 | 4 | water | Mv | Yes | 1 | 1 | 1 | 0 |
| D3 | 6 | water | Mv | Yes | 1 | 0 | 0 | 0 |
| D3 | 7 | water | Mv | Yes | 1 | 0 | 0 | 0 |
| D3 | 8 | water | Mv | Yes | 1 | 0 | 0 | 0 |
| D3 | 1 | fungicide | Ev | No | 0 | 0 | 0 | 0 |
| D3 | 2 | fungicide | Ev | No | 0 | 0 | 0 | 0 |
| D3 | 4 | fungicide | Ev | No | 0 | 0 | 0 | 0 |
| D3 | 7 | fungicide | Ev | Yes | 1 | 0 | 0 | 0 |
| D3 | 9 | fungicide | Ev | No | 0 | 0 | 0 | 0 |
| D3 | 10 | fungicide | Ev | No | 0 | 0 | 0 | 0 |
| D3 | 2 | water | Ev | Yes | 0 | 0 | 0 | 0 |
| D3 | 3 | water | Ev | No | 0 | 0 | 0 | 0 |
| D3 | 6 | water | Ev | No | 0 | 0 | 0 | 0 |
| D3 | 7 | water | Ev | No | 0 | 0 | 0 | 0 |
| D3 | 9 | water | Ev | No | 0 | 0 | 0 | 0 |
| D3 | 10 | water | Ev | No | 0 | 0 | 0 | 0 |
| D4 | 2 | water | Mv | Yes | 1 | 0 | 0 | 0 |
| D4 | 10 | water | Mv | Yes | 1 | 0 | 0 | 0 |
| D4 | 3 | fungicide | Ev | No | 0 | 0 | 0 | 0 |
| D4 | 5 | fungicide | Ev | No | 0 | 0 | 0 | 0 |
| D4 | 6 | fungicide | Ev | No | 0 | 0 | 0 | 0 |
| D4 | 9 | fungicide | Ev | No | 0 | 0 | 0 | 0 |
| D4 | 2 | water | Ev | No | 0 | 0 | 0 | 0 |
| D4 | 3 | water | Ev | No | 0 | 0 | 0 | 0 |
| D4 | 5 | water | Ev | No | 0 | 0 | 0 | 0 |
| D4 | 8 | water | Ev | Yes | 0 | 0 | 0 | 0 |
| D4 | 10 | water | Ev | Yes | 0 | 0 | 0 | 0 |
