# Supplementary material for: Emerging fungal pathogen of an invasive grass: Implications for competition with native plant species
Source: PLoS One. 2021 Mar 1;16(3):e0237894. doi: 10.1371/journal.pone.0237894 (PMC7920361; doi:10.1371/journal.pone.0237894)
Supplement: Attachment — Submitted filename: Response to Reviewers_.docx [file pone.0237894.s005.docx]

**Richard A Wilson
Academic Editor
PLOS ONE

Journal Requirements:**

**When submitting your revision, we need you to address these additional requirements.**

**1. Please ensure that your manuscript meets PLOS ONE's style requirements, including those for file naming. The PLOS ONE style templates can be found at**

[**https://journals.plos.org/plosone/s/file?id=wjVg/PLOSOne_formatting_sample_main_body.pdf**](https://journals.plos.org/plosone/s/file?id=wjVg/PLOSOne_formatting_sample_main_body.pdf) **and**

[**https://journals.plos.org/plosone/s/file?id=ba62/PLOSOne_formatting_sample_title_authors_affiliations.pdf**](https://journals.plos.org/plosone/s/file?id=ba62/PLOSOne_formatting_sample_title_authors_affiliations.pdf)

We renamed the supplementary files according to the file name guidelines and formatted the sub-section headings in the Materials and methods and Discussion according to guidelines. We removed the period after “Fig.” We changed “Supporting Information Captions” to sentence case. We named text files as described above.

**2. In your Methods section, please provide additional location information of the collection sites, including geographic coordinates for the data set if available.**

 We added coordinates for the seed collection site:

“We used *Microstegium* seeds collected from Big Oaks National Wildlife Refuge (BONWR; 38.9365, -85.4148) in 2015…” (Lines 118–119)

**3. In your Methods section, please provide additional information regarding the permits you obtained for the work. Please ensure you have included the full name of the authority that approved the field site access and, if no permits were required, a brief statement explaining why.**

We added permit information:

“*Bipolaris gigantea* isolations were in accordance with the United States Department of Agriculture Animal and Plant Health Inspection Service Plant Protection and Quarantine (USDA APHIS PPQ) permit no. PP526P-18-01688. Collections from BONWR were performed under a special use permit administered by the United States Fish and Wildlife Service.” (Lines 169–172)

**4. We note that you have stated that you will provide repository information for your data at acceptance. Should your manuscript be accepted for publication, we will hold it until you provide the relevant accession numbers or DOIs necessary to access your data. If you wish to make changes to your Data Availability statement, please describe these changes in your cover letter and we will update your Data Availability statement to reflect the information you provide.**

Information for the data and code package have been updated following revisions and can be accessed with the link below. This is the staging platform for the Environmental Data Initiative and we will move the package to the public-facing platform upon acceptance.

https://portal-s.edirepository.org/nis/mapbrowse?scope=edi&identifier=63&revision=2 **[Note: HTML markup is below. Please do not edit.]

Reviewers' comments:

Reviewer's Responses to Questions**

**Comments to the Author

1. Is the manuscript technically sound, and do the data support the conclusions?

The manuscript must describe a technically sound piece of scientific research with data that supports the conclusions. Experiments must have been conducted rigorously, with appropriate controls, replication, and sample sizes. The conclusions must be drawn appropriately based on the data presented.**

**Reviewer #1: No**

**Reviewer #2: No**

**Reviewer #3: Yes**

**2. Has the statistical analysis been performed appropriately and rigorously?**

**Reviewer #1: Yes**

**Reviewer #2: Yes**

**Reviewer #3: Yes**

**3. Have the authors made all data underlying the findings in their manuscript fully available?

The** [**PLOS Data policy**](http://www.plosone.org/static/policies.action#sharing) **requires authors to make all data underlying the findings described in their manuscript fully available without restriction, with rare exception (please refer to the Data Availability Statement in the manuscript PDF file). The data should be provided as part of the manuscript or its supporting information, or deposited to a public repository. For example, in addition to summary statistics, the data points behind means, medians and variance measures should be available. If there are restrictions on publicly sharing data—e.g. participant privacy or use of data from a third party—those must be specified.**

**Reviewer #1: Yes**

**Reviewer #2: Yes**

**Reviewer #3: No**

**4. Is the manuscript presented in an intelligible fashion and written in standard English?

PLOS ONE does not copyedit accepted manuscripts, so the language in submitted articles must be clear, correct, and unambiguous. Any typographical or grammatical errors should be corrected at revision, so please note any specific errors here.**

**Reviewer #1: Yes**

**Reviewer #2: Yes**

**Reviewer #3: Yes**

**5. Review Comments to the Author

Please use the space provided to explain your answers to the questions above. You may also include additional comments for the author, including concerns about dual publication, research ethics, or publication ethics. (Please upload your review as an attachment if it exceeds 20,000 characters)**

**Reviewer #1: The aim of this study was to determine how the pathogen (Bipolaris) and invasive Microstegium impacted competition among native and invasive plant.

Major comment:

1. It seems very strange that native plants would only develop symptoms in the presence of invasive Microstegium. This deserve some explanation. How does an invasive plant make native plants more susceptible to pathogen?**

Good question. We added text to the Discussion to hypothesize mechanisms that could explain this result:

“Fungal lesions were observed on native plants grown with *Microstegium*, perhaps because *Microstegium* biomass altered the microclimate of the pots (e.g., increased humidity), which can be more suitable for lesion formation [56]. In addition, transmission from *Microstegium* to native plants may have maintained infections. Indeed, *B. gigantea* transmission from more competent host species to less competent host species has been inferred from field observations [37].” (Lines 411–416)

**2. According to the authors, L177: ‘There were too few native plants with lesions. With that being said, how can the authors speculate the following at Line 268: ‘These results suggest that disease caused by Bipolaris may alter the community composition of native species. This claim seems farfetched and unsupported, since ‘there were too few native plants with lesions.**

While we wrote this interpretation in light of the effects of *B. gigantea* inoculation on native plant biomass, we understand why it is confusing and out of the scope of our experiment.

We revised the text in the Discussion to a more conservative interpretation:

“These results suggest that *B. gigantea* may differentially affect native plant species in competition with *Microstegium* through species-specific responses to *B. gigantea* exposure.” (Lines 339–341)

In addition, we added a paragraph to the Discussion describing how the data collected in this experiment contributes to our understanding of competition and its implications for plant community composition.

“Studies on disease-mediated competition between invasive and native plant species indicate that infection of invasive plants can contribute to either native plant persistence or recovery [14,15,34,54]. However, in our experiment, inoculation with *Bipolaris* did not modify the effect of *Microstegium* density on the three native species relative to the mock inoculation control, suggesting that competitive effects of *Microstegium* on native species are likely to be consistent in the presence or absence of low levels of disease incidence. Our experimental design captured two components of native species’ competitive ability: their growth in the absence of competition and their biomass responses to changes in *Microstegium* density (i.e., interspecific competition coefficients) [41,42]. To better characterize the competitive ability of the native species, it is also necessary to estimate their biomass responses to changes in their own density (i.e., intraspecific competition coefficients) [41,42]. Because *B. gigantea* inoculation had unique effects on the growth of the three native species, it may also uniquely affect their per capita impacts on competitor growth, altering their intraspecific competition coefficients. If, however, *B. gigantea* does not affect the intraspecific competition coefficients of the native species, we would expect low levels of *B. gigantea* exposure to increase the competitive ability of *Dichanthelium* in interactions with *Microstegium*, decrease the competitive ability of *Elymus*, and to have no effect on the competitive ability of *Eragrostis*, potentially leading to shifts in the relative abundances of native species.” (Lines 344–361)

**3. The authors report disease incidence at Line 209 -213, and then provide an explanation that is not intuitive.**

We agree that the description of the results was unclear here. We revised this text in the Results:

“We observed *Bipolaris*-like lesions on *Microstegium* leaves in 94% of pots in which *Microstegium* was planted and inoculated. The average *Microstegium* disease incidence in low-density pots (i.e., two *Microstegium* plants) was 8% (95% CI: 6%–11%). The species identity of the native plant in low density pots did not significantly affect *Microstegium* disease incidence (Table 1). *Microstegium* disease incidence was constant across the *Microstegium* density gradient when *Elymus* was present (Fig 2B). However, *Microstegium* disease incidence decreased by four percentage points (95% CI: -7% – -2%) and five percentage points (95% CI: -7% – -3%) when 100 *Microstegium* were grown with *Dichanthelium* and *Eragrostis*, respectively, relative to pots with two *Microstegium* plants and the same native species (Fig 2A, C). The total number of leaves per pot increased across the *Microstegium* density gradient while the number of leaves with lesions increased more slowly or not at all (Fig S1).” (Lines 250–260)

For our response to the unintuitive explanation, please see comment #7.

**4. The results are reported as ‘disease incidence’, but then authors explain disease severity. Disease incidence is should be per plant/individual. Disease severity should be per leaves.**

We reported the occurrence of disease without regard for the size of the lesions, which is the definition of disease incidence in the field of plant pathology. Nutter et al. 2006 provides this definition: “Disease incidence is defined as the number of sampling units (e.g., leaflets, leaves, stems, tillers, whole plants, seeds, etc.) that are diseased (expressing symptoms), divided by the total number of sampling units sampled and assessed (Nutter et al., 1991; Nutter, 1997b, 1999).”

We added text for this definition and this reference to the Materials and methods:

“To assess disease incidence (i.e., the percentage of leaves per pot with at least one lesion [45]) 14 days after inoculation, we recorded the number of *Microstegium* leaves with one or more *Bipolaris*-like lesions (Fig 1C) and the total number of leaves for three *Microstegium* plants per pot (or two plants for the pots with only two).” (Lines 175–178)

Ref 45: Nutter FW, Esker PD, Netto RAC. Disease assessment concepts and the advancements made in improving the accuracy and precision of plant disease data. Eur J Plant Pathol. 2006;115: 95–103. doi:10.1007/s10658-005-1230-z

We performed a search in Google Scholar and were unable to locate a reference in which the goal was to clarify terminology and the proportion of leaves with lesions was called severity.

**5. It is safe to assume that the authors did not perform preliminary studies to determine if the pathogen could infect the native plants used in this study. L89 -93, the author explicitly mentions that because the native plants occur with the invasive, they assumed the natives were susceptible. The study would have actually benefited from screening, testing, and using susceptible natives. This is an interesting pilot study.**

We did not perform preliminary studies on susceptibility to *Bipolaris gigantea* for *Dichanthelium clandestinum* and *Eragrostis spectabilis* because we felt that the question of how disease mediates the competitive effect of *Microstegium* on different native species is relevant whether or not the native species are susceptible to infection. We revised multiple aspects of the manuscript to better explain this issue.

a. We revised the title to: “Emerging fungal pathogen differentially affects three native plant species that compete with an invasive grass”
b. We revised the Abstract to better explain the question:

“Infectious diseases and invasive species can be strong drivers of biological systems that may interact to shift plant community composition. For example, disease can modify resource competition between invasive and native species. Invasive species tend to interact with a diversity of native species, and it is unclear how native species differ in response to disease-mediated competition with invasive species. Here, we quantified the biomass responses of three native North American grass species (*Dichanthelium clandestinum*, *Elymus virginicus*, and *Eragrostis spectabilis)* to disease-mediated competition with the non-native invasive grass *Microstegium vimineum*.” (Lines 18–25)

c. We revised the Abstract to better explain the broader implications:

“Thus, *B. gigantea* had species-specific effects on native plant competition with *Microstegium* through species-specific biomass responses to *B. gigantea* inoculation, but not through modified responses to *Microstegium* density. Our results suggest that disease may uniquely modify competitive interactions between invasive and native plants for different native plant species.” (Lines 34–38)

d. We revised the Introduction to emphasize disease-mediated competition:

“However, the relevant guild of native species in natural communities is often diverse and species vary in their susceptibility to pathogen infection [15], making it unclear whether results from studies of disease-mediated competition with one native species apply to the broader native community.” (Lines 52–55)

“*Bipolaris gigantea* may alter the competitive ability of *Microstegium* or the ability of these native species to compete with *Microstegium*. Competitive ability depends on the fitness of species in the absence of competition and their responses to changes in intraspecific and interspecific competitor densities (i.e., competition coefficients) [41,42]. Here we investigated how *B. gigantea* inoculation affected the competitive ability of the three native perennial grass species in greenhouse experiment by measuring their competition-free biomass and responses to *Microstegium* density.” (Lines 100–106)

e. We changed the order of the Discussion to put disease-mediated competition at the beginning and added sub-headings.
f. We changed terminology throughout from “direct” and “indirect” effects of disease on the native species to “growth in the absence of competition” and “biomass responses to increasing densities of *Microstegium*”, respectively (e.g., Lines 29–29, 106)
g. We changed terminology throughout from “pathogen inoculation” to “*B. gigantea* inoculation” because we are confident in our methods for isolating *B. gigantea*, but less confident that it has “pathogenic” effects on each species in the study (e.g., Lines 35, 104).

We revised the text about co-occurrence of the native species with *Microstegium* to clarify that we do not consider this evidence that the species are susceptible to *B. gigantea* infection.

We added text to better organize the references, including our prior study on *Elymus virginicus* susceptibility to *B. gigantea* and information about the susceptibility of the other two native species:

“*Dichanthelium clandestinum* L. Gould (syn. *Panicum clandestinum* L.; deer-tongue grass, hereafter *Dichanthelium*), *Elymus virginicus* L. (Virginia wild rye, hereafter *Elymus*), and *Eragrostis spectabilis* (Pursh) Steud (purple lovegrass, hereafter *Eragrostis*) are perennial grass species that are native to the U.S. and co-occur with *Microstegium* [29,34]. *Bipolaris gigantea* can infect, produce lesions on, and produce spores on *Elymus virginicus* [35]. *Bipolaris gigantea* infections have been observed on *Dichanthelium clandestinum* in the field, but it may not be a competent host for spore production [37]. At least three species in the genus *Eragrostis* are susceptible to *B. gigantea* infection [37–39] and closely related plant species are more likely to share pathogens than distantly related species [30], suggesting that *Eragrostis spectabilis* may also be susceptible to *B. gigantea*.” (Lines 89–98)

**6. An interesting result that might be worthy of explaining is the different outcome that Bipolaris had on 3 different native plants.**

Yes, we agree that the variation in responses among the native species is interesting. We modified and added text in the Discussion to better address this topic:

“The three native species in our experiment showed unique biomass responses to *B. gigantea* inoculation in the absence of *Microstegium* competition. The range of *B. gigantea* inoculation effects on biomass, from negative (on *Elymus*) to positive (on *Dichanthelium*) is consistent with the theory that plant-microbe interactions can vary from mutualism to parasitism depending on context, such as environmental conditions and host identity [59,60]. For example, infection with Cucumber mosaic virus increased the biomass and seed weight of one *Arabidopsis thaliana* genotype while it reduced the biomass and seed weight of another genotype relative to a mock inoculation control [63]. Our results suggest that *B. gigantea* could increase aboveground growth for some host species (e.g., through compensatory growth or re-allocation of resources [22,63]) and suppress the aboveground growth of other species. Studies encompassing a broader range of environmental conditions and host diversity are needed to better predict when *B. gigantea* will have positive, negative, or neutral effects on host biomass and other traits.” (Lines 391–403)

We also added text to the Introduction to prepare the reader for these potential outcomes:

“We were uncertain about how *B. gigantea* would affect native species biomass, but acknowledged that a range of outcomes were possible given interspecific variation in host-pathogen interactions [29–31], including decreased, increased (e.g., through compensatory growth), and no change in biomass.” (Lines 106–109)

**7. According to table S1, Microstegium disease incidence decreased as microstegium density increased. As mentioned before the explanation needs more insight.**

Yes, *Microstegium* disease incidence decreased as *Microstegium* density increased. We edited the text describing this result to make it clearer (see response to comment # 3) and provided a possible explanation in the Discussion:

“For example, disease incidence on *Microstegium* decreased as *Microstegium* densities increased, which is likely because the single *B. gigantea* inoculation infected a relatively constant number of leaves regardless of *Microstegium* density, leading to lower percentages of leaves with lesions at higher densities.” (Lines 374–377)

**8. As for the results section, the authors do not provide a P-values, but report signify**

**cance via supplementary table, where 95% CI does not intersect zero.**

Yes, we used the 95% credible intervals of the posterior distributions to determine statistical significance. So that readers do not have to refer to supplementary tables, we added 95% credible intervals to the Results text and moved the supplementary tables to the main text. For results from models with logit links, we transformed the estimates and 95% CI reported in the text to percentages (e.g., response to comment #3). We also added explanatory text to the Materials and methods:

“We calculated the mean and 95% highest posterior density interval (hereafter, “credible interval”) of back-transformed (from logit to percentage) model estimates using the mean_hdi function in the tidybayes package [50].” (Lines 215–217)

**9. Truthfully, this is my first time reading a manuscript without a reported p value. The readers will find it odd that the results section does not mention a p value. values need to be included**

We understand that many traditional analyses include p-values, however, we chose to use Bayesian analysis for the statistical models in this study because the brms package makes it straightforward to estimate treatment effects on the parameters of non-linear functions, such as the one we used for native plant biomass. Credible intervals from the posterior distributions of Bayesian analyses and other alternatives to P-values (e.g., ΔAIC) are common in ecological studies (please see recent examples from PLoS One below).

To make our results more accessible to readers who are less familiar with this area of the literature, we added a definition of credible intervals and two references with additional information:

“We report a model coefficient as statistically significant if its 95% credible interval (“CI”, i.e., 95% probability that this interval of the posterior distribution contains the true estimate value) omits zero [51,52].”.

Ref 51: McElreath R. Ch. 3: Sampling the Imaginary. Statistical Rethinking: A Bayesian Course with R Examples. Boca Raton, FL, USA: Taylor & Francis Group; 2015. pp. 49–70. doi:10.1201/9781315372495-3

Ref 52: Ellison AM. Bayesian inference in ecology. Ecol Lett. 2004;7: 509–520. doi:10.1111/j.1461-0248.2004.00603.x

Also, here are some recent PLoS One publications that report credible intervals to indicate statistical significance in the results:

Junaedi DI, McCarthy MA, Guillera-Arroita G, Catford JA, Burgman MA. Traits influence detection of exotic plant species in tropical forests. PLoS One. 2018;13: 1–15. doi:10.1371/journal.pone.0202254

Wang H, Snapp SS, Fisher M, Viens F. A Bayesian analysis of longitudinal farm surveys in Central Malawi reveals yield determinants and site-specific management strategies. PLoS One. 2019;14: 1–17. doi:10.1371/journal.pone.0219296

Larson MD, Levri EP, Huzurbazar S V., Greenwood DJ, Wise KL, Krist AC. No evidence for a dilution effect of the nonnative snail, Potamopyrgus antipodarum, on native snails. PLoS One. 2020;15: 6–12. doi:10.1371/journal.pone.0239762

**Minor Comments

10. Line 21: After mentioning ‘North American grass species. I recommend adding a parenthesis and naming the native grass species.**

We made this change:

“Here, we quantified the biomass responses of three native North American grass species (*Dichanthelium clandestinum*, *Elymus virginicus*, and *Eragrostis spectabilis)* to disease-mediated competition with the invasive grass *Microstegium vimineum*.” (Lines 22–25)

**11. Line 22: Hard stop, add period after Bipolaris gigantea. Now make a new sentence.
i.e This foliar pathogen has recently emerged in populations of the invasive grass Microstegium vimineum, causing leaf spot disease**

We made this change:

“The foliar fungal pathogen *Bipolaris gigantea* has recently emerged in *Microstegium* populations, causing a leaf spot disease that reduces *Microstegium* biomass and seed production*.*” (Lines 25–27)

**12. Line 28: Remove: “inoculation treatment”. Replace with “pathogen outcome”**

Thank you. We considered the suggestion “pathogen outcome,” but this could be interpreted to mean we tracked the fate of the pathogen (i.e., outcome of the pathogen), when the focus of our study was the resulting disease incidence and host effects.

We clarified the text in response to the comment by changing “inoculation treatment” to “*Bipolaris gigantea* inoculation”:

“*Bipolaris gigantea* inoculation did not, however, affect *Microstegium* biomass or mediate the effect of *Microstegium* density on native plant biomass.” (Lines 32–34)

**13. Line 29: Remove: “suggesting that disease had no indirect effects through altered competition’
ADD: “suggesting that pathogen outcome did not alter competition”:**

Thank you for this suggestion. This text was removed during revisions. **14. Line 38: Replace “afflict” with ‘perturb’**

We made this change (“affect”, Reviewer #3):

“Both plant invasions and infectious diseases can affect natural plant communities by reducing plant diversity and biomass production [1–4].” (Lines 41–42)

**15. Line 41 – 42: This is unclear because plant invasions are a spatiotemporal process that stems from (1) transport, (2) colonization, (3) establishment, and (4) landscape spread(Theoharides and Dukes, 2007), but It appears as if the author is using plant introduction synonymous with plant invasion. How is community structure being maintained, if an invasion suggests altered community structure**

We clarified the meaning of this sentence by rephrasing it:

“Invasive plants can negatively impact native plant communities through competition [6] and diseases may increase, decrease, or have no net effect on invasive plant impacts [7,8].” (Lines 44–46)

**16. Line 43: This is unclear: “suppressing native species that would either driver on its own”.**

This text was removed in the revision described in the response to comment #15. **17. Line 44: This sentence is unclear. Perhaps avoid starting a sentence with a conjunction (i.e “Which”), and it will be easier for the reader to understand**

We agree and we revised this sentence:

“The responses of both the invasive species and competing native species to infection can determine which of these outcomes occurs [9,10].” (Lines 46–47)

**18. Line 45 – 47: This line can be more precise. Also, it seems untimely because at Line 41 -44 an example is given of ‘disease and plant invasion’. Line 45 – 47 is saying the same thing again. Only difference is in Line 41 -44 the reader is told the potential outcome of ‘disease and plant invasion’, and in Line 45 – 47 the outcome is vague but with more references.**

We revised this sentence to clarify that previous tests have been between a single native species and a single invasive species, which has provided insights but may not be applicable to all relevant co-occurring native species:

“In empirical and theoretical tests of disease-mediated competition between a single native plant species and a single invasive plant species, disease has both increased [12,13] and decreased [14,15] impacts of the invasive species. However, the relevant guild of native species in natural communities is often diverse and species vary in their susceptibility to pathogen infection [16], making it unclear whether results from studies of disease-mediated competition with one native species apply to the broader native community.” (Lines 49–55)

**19. Line 62: Replace: “occurred” with “was reported”**

We made this change:

“If disease disproportionately impacts invasive species, reductions in growth and resource uptake may release native species from competition, which was reported when a powdery mildew fungus infected invasive garlic mustard (*Alliaria petiolata*) [15].” (Lines 70–73)

**20. Line 70: Formally introduce the pathogen. Mentioning that an anonymous pathogen suppresses growth and reproduction and providing two reference seems to be a stating the obvious. In general, there are some references in this section, without the thought or example being fully defines**

We added text to formally introduce the pathogen in the Introduction:

**“**Over the last two decades, *Microstegium* populations have acquired fungal leaf spot diseases caused by species in the genus *Bipolaris* that reduce its biomass and seed production [29,34]. *Bipolaris gigantea* (Heald & F.A. Wolf) B. Lane, Stricker, M.E. Sm., S.L. Flory & Harmon is a common pathogen of *Microstegium* [34] that causes zonate leaf spots with dark brown margins [35] and likely disperses via wind and splashing [36], but with restricted distances due to its large spore size [35,37].” (Lines 82–88)

**21. Line 92 - 93: Pathogen is formally mentioned, but there isn’t any mention of symptoms, or how it spreads. Also, the authors mention that the native grass species maybe to susceptible to Bipolaris, but due to the nature of this study, it seems a preliminary test to confirm susceptibility would have been helpful**

We added text to describe the symptoms and what is known about its spread (see response to comment #20). We also added text to better describe the known susceptibility of each species (see response to comment #5).

**22. L209 -213: A disease incidence is presence or absence of disease on a particular plant. However, the author seems to report disease incidence, and then gives a disease severity interpretation (i.e leaves with lesions)**

We disagree with the contention. Incidence can be expressed at any level: per cell, per leaf, per plant, per field, per state, etc. Please see response to comment #4.

**23. L211 -213: Interpretation should be explained in the discussion**

We removed this text from the Results and added an interpretation to the Discussion. Please see responses to comments #3 and #7 for text.

**Reviewer #2: Manuscirpt by Kendig et al investigates the impact of fungal pathogen Bipolaris on one invasive grass Microstegium vimineum in North America as well as on three native grasses, Dichanthelium clandestinum Elymus virginicus, and Eragrostis spectabilis. In addition their aim was to test how pathogen impacts on the competition between the plant species. The experimental design includes the three separate native grass species treatments alone and in different densities of invasive grass and Bipolaris inoculation and mock inoculations in greenhouse. The authors find that the pathogen has minor effect on the biomass of the native grasses in the absence of the invasive grass but the invasive grass has overall larger negative impact on the native grasses than the pathogen. However, there are major methodological issues that must be addressed before being able to evaluate the manuscript further:**

**Main problems:**

**1. First, the authors do not soundly establish that the three Native grasses are susceptible for Bipolaris. This should be done by either referencing previous literature that explicitly shows that, in ref 24 Flory et al 2014 show that Elymus is susceptible to some strains of Bipolaris but not all of them. I did not find the other two grass species in the refs 24, 27, 32, 33. Or, alternatively by performing a laboratory trial on with the strain in concern and with all the native species and then using the strain in the experiment. Or, by taking the leaf samples from the plants used in the experiment, performing microscopy, growing the pathogen isolate on growth media plates and identification from them, or by PCR from the infected leaf tissue. None of these approaches is currently taken.**

We appreciate that susceptibility of native species is a major concern, and it was also brought up by Reviewer #1. We revised the manuscript to emphasize the research question, “How does disease modify the competitive effect of *Microstegium* on three native species?”. We provide text excerpts in our response to Reviewer #1 comment #5 to demonstrate these revisions. We believe that with this focus, it is not central whether native species are susceptible or not. For example, disease can modify the competitive impact of the invasive species on a resistant native species.

In addition, we clarified the references providing information about native plant susceptibility by revising the sentence, including the full name of the pathogen, and including synonyms for the plants.

“*Bipolaris gigantea* (Heald & F.A. Wolf) B. Lane, Stricker, M.E. Sm., S.L. Flory & Harmon is a common pathogen of *Microstegium* [34] that causes zonate leaf spots with dark brown margins [35] and likely disperses via wind and splashing [36], but with restricted distances due to its large spore size [35,37]. *Dichanthelium clandestinum* L. Gould (syn. *Panicum clandestinum* L.; deer-tongue grass, hereafter *Dichanthelium*), *Elymus virginicus* L. (Virginia wild rye, hereafter *Elymus*), and *Eragrostis spectabilis* (Pursh) Steud (purple lovegrass, hereafter *Eragrostis*) are perennial grass species that are native to the U.S. and co-occur with *Microstegium* [29,34]. *Bipolaris gigantea* can infect, produce lesions on, and produce spores on *Elymus virginicus* [35]. *Bipolaris gigantea* infections have been observed on *Dichanthelium clandestinum* in the field, but it may not be a competent host for spore production [37]. At least three species in the genus *Eragrostis* are susceptible to *B. gigantea* infection [37–39] and closely related plant species are more likely to share pathogens than distantly related species [30], suggesting that *Eragrostis spectabilis* may also be susceptible to *B. gigantea*.” (Lines 84–98)

**2. Second, the authors have not discussed the possibility that the symptoms in the study plants are caused by another pathogen, potentially from seed (again, lack of any detection methods in the experiment).**

We recognize and appreciate the concern voiced and have made several changes below. However, the inclusion of mock-inoculated controls, and the direct comparison of inoculated vs. mock-inoculated treatments limit the effects these potential sources of error could have on our results. The only difference between the inoculated and mock inoculated treatments was the application of the *B. gigantea* inoculum. Additionally, the symptoms observed were exactly those previously shown through the completion of Koch’s Postulates to be caused by this pathogen on the *Microstegium* host (ref #35 in the main text).

We added text to the Materials and methods to demonstrate that lesions observed on leaves were unlikely to come from contaminant pathogens. We already included text describing sterilization of soil and pots.

“Prior to the experiment, seeds of each species were planted in a greenhouse and seedlings developed no lesions, suggesting that lesions caused by *B. gigantea* inoculation were unlikely to be confused with lesions caused by potential seedborne pathogens.” (Lines 121–124)

“To assess disease incidence (i.e., the percentage of leaves per pot with at least one lesion [45]) 14 days after inoculation, we recorded the number of *Microstegium* leaves with one or more *Bipolaris*-like lesions (Fig 1C) and the total number of leaves for three *Microstegium* plants per pot (or two plants for the pots with only two). No other types of lesions were observed on the plants.” (Lines 175–179)

In addition, we added text to the Discussion to address the possibility of contaminant pathogens and describe why our interpretation of the results is still valid:

“Although we did not test plants for infection with *B. gigantea* infection following the experiment, our results indicate that observed lesions and biomass effects were likely due to the *B. gigantea* inoculation treatment. Only a single pot in the mock inoculation treatment exhibited disease symptoms while 94% of *B. gigantea*–inoculated pots with *Microstegium* (*n* = 48) displayed disease symptoms. In addition, a relatively high number of leaves in the one mock-inoculated pot had lesions. These two results suggest that the pot was inadvertently inoculated when treatments were applied. An alternative explanation is that seeds harboured pathogenic fungi or contaminants were introduced to the one mock-inoculated pot and none of the other mock-inoculated pots. While either is a possibility, the latter does not explain the much higher percentage of inoculated pots with lesions relative to mock-inoculated pots. In addition, *B. gigantea* can co-occur with other pathogens in the field (Adhikari et al., unpublished data). Therefore, infection of plants in our experiment that is not confounded with the inoculation treatment does not negate our ability to evaluate the effects of the inoculated *B. gigantea* strain on competition between native plants and *Microstegium*. Future efforts that aim to better characterize host-pathogen interactions between *Microstegium* or native plant species and *Bipolaris* fungi could confirm infection by attempting to re-isolate the fungus after inoculation.” (Lines 441–456)

**3. Third, Bipolaris infection found in the pathogen treated and mock inoculated plants (l. 158-160) may be seedborne. This should have be established by using pathogen free seed, testing all plant material after germination prior experiment for Bipolaris by PCR, or by testing mock inoculated plants with PCR in the end of the experiment.**

The text we added in response to comment #2 describes why we believe it is unlikely that lesions observed on leaves were from a seedborne pathogen. While revising the manuscript, we were careful to describe the results in response to the *B. gigantea* inoculation treatment. Because we are confident in the *B. gigantea* isolation procedure and because we administered a mock inoculation control, we believe it is fair to assess results in the context of this treatment. Any background level of seedborne or other disease symptom is accounted for in the experimental design and statistical analysis (along with other sources of experimental error). Pathogen free seed is not feasible nor required.

We added information from a separate field experiment (which is in preparation for publication) to the Materials and methods to provide readers with a quantitative measure of the efficacy of our approach:

“*Bipolaris gigantea* association with foliar lesions can be assessed by using microscopy to identify conidiophores on leaves after incubation [35]. The absence of *B. gigantea* conidiophores in lesions, however, does not confirm that it is not the causal agent. In leaf samples collected from BONWR in 2018 and 2019, 67% of *Microstegium* samples (*n* = 238) and 48% of *Elymus* samples (*n* = 65) that had *Bipolaris*-like lesions also had *B. gigantea* conidiophores identifiable by microscopy. In addition, 28% of *Microstegium* samples (*n* = 29) and 1% of *Elymus* samples (*n* = 106) that did not have *Bipolaris*-like lesions had *B. gigantea* conidiophores identifiable by microscopy (Adhikari et al., unpublished data). Therefore, *Bipolaris*-like lesions are commonly associated with *B. gigantea* infection and it is less common for leaves to be infected without lesions.” (Lines 186–195)

**Reviewer #3: The research article ‘Emerging fungal pathogen on an invasive grass differentially affects native species’ documents a well-planned experimental examination of the effects on three native grasses (Dichanthelium, Elymus and Eragrotis) of an invasive grass, Microstegium vimineum, in the presence of a fungal pathogen, Bipolaris gigantea. The manuscript is well written. Results show that the density of the invasive grass has effects on the native grasses that overwhelms any effect of the presence of the pathogen on either the invasive grass itself or the native grasses. Some differential effects of the fungus on the native grasses is observed, however infection patterns across individuals suggest that the inoculation protocol failed at some level – indeed the authors concede that conidial concentrations of the inoculum were low, perhaps by several orders of magnitude.**

**1. I believe more needs to be said about the efficacy of the inoculation procedure.**

We added a section to the Discussion titled Limitations that includes a description of the efficacy of the inoculation procedure (text previously present in the Discussion) and additional text about the possibility of contamination by another pathogen (Response to Reviewer #2 Comment #2).

“Experimentally suppressing *Bipolaris* infection using fungicide in the field increased *Microstegium* biomass by 33–39% [29,34], suggesting substantial effects of severe disease symptom development. However, despite using a pathogenic *Bipolaris* isolate in our experiment, inoculation caused relatively low levels of disease incidence (relative to approximately 40% of leaves with lesions documented in the field site where infected litter was collected, Kendig et al. unpublished data), which had no effect on *Microstegium* biomass relative to the mock inoculation control. While such results could be explained by *Microstegium* tolerance or compensatory growth [22,23], it is more parsimonious to assume that *B. gigantea* exposure was below levels experienced in the field. Pathogen transmission and disease incidence depend on the favorability and duration of environmental conditions and the inoculum load [63,64]. Our experiment relied on a single inoculation and extended incubation; however, field conditions that result in cycles of leaf wetness events (e.g., dew or precipitation) can enhance fungal infection [63] and promote multiple disease cycles throughout the growing season. The concentration of *Bipolaris* conidia in our experimental inoculations was limited by the number of conidia we could harvest from agar plates in the lab and was relatively low (15,000 conidia/ml compared to e.g., 10^5^ conidia/ml [64]). While the conditions for leaf wetness and conidia suspension concentration likely limited the possible extent of disease incidence, they may reflect initial disease dynamics in the field, which is consistent with the age of plants we used in the experiment.

Although we did not test plants for infection with *B. gigantea* infection following the experiment, our results indicate that observed lesions and biomass effects were likely due to the *B. gigantea* inoculation treatment. Only a single pot in the mock inoculation treatment exhibited disease symptoms while 94% of *B. gigantea*–inoculated pots with *Microstegium* (*n* = 48) displayed disease symptoms. In addition, a relatively high number of leaves in the one mock-inoculated pot had lesions. These two results suggest that the pot was inadvertently inoculated when treatments were applied. An alternative explanation is that seeds harboured pathogenic fungi or contaminants were introduced to the one mock-inoculated pot and none of the other mock-inoculated pots. While either is a possibility, they do not explain the much higher percentage of inoculated pots with lesions relative to mock-inoculated pots. In addition, *B. gigantea* can co-occur with other pathogens in the field (Adhikari et al., unpublished data). Therefore, infection of plants in our experiment that is not confounded with the inoculation treatment does not negate our ability to evaluate the effects of the inoculated *B. gigantea* strain on competition between native plants and *Microstegium*. Future efforts that aim to better characterize host-pathogen interactions between *Microstegium* or native plant species and *Bipolaris* fungi could confirm infection by attempting to re-isolate the fungus after inoculation.” (Lines 421–456)

**2. A more thorough discussion concerning the differential direct effects of the fungus on the native grasses that were infected would be welcome.**

We modified and added text to the Discussion to better address differential effects on native species:

“It is likely that pathogen amplification by invasive plants will have distinct effects on different native species [20,31]. The three native species in our experiment showed unique biomass responses to *B. gigantea* inoculation in the absence of *Microstegium* competition. The range of *B. gigantea* inoculation effects on biomass, from negative (on *Elymus*) to positive (on *Dichanthelium*) is consistent with the theory that plant-microbe interactions can vary from mutualism to parasitism depending on context, such as environmental conditions and host identity [59,60]. For example, infection with Cucumber mosaic virus increased the biomass and seed weight of one *Arabidopsis thaliana* genotype while it reduced the biomass and seed weight of another genotype relative to a mock inoculation control [63]. Our results suggest that *B. gigantea* could increase aboveground growth for some host species (e.g., through compensatory growth or re-allocation of resources [22,63]) and suppress the aboveground growth of other species. Studies encompassing a broader range of environmental conditions and host diversity are needed to better predict when *B. gigantea* will have positive, negative, or neutral effects on host biomass and other traits.” (Lines 390­–403)

**Detailed comments**

**3. Line 38. ‘affect’ not ‘afflict’**

We changed this text:

“Both plant invasions and infectious diseases can affect natural plant communities by reducing plant diversity and biomass production [1–4].” (Lines 41–42)

**Line 88. infections ‘with’ species not ‘from’**

We changed this text:

“Over the last two decades, *Microstegium* populations have acquired fungal leaf spot diseases caused by species in the genus *Bipolaris* that reduce its biomass and seed production [29,34].” (Lines 82–84)

**Line 101. plastics ‘bags’ not ‘bag’**

This text was removed during revisions.

**Line 138. ‘hemocytometer’ (or haemocytometer) not ‘hemacytometer’**

We changed this text:

“The resulting conidia suspension was filtered through a layer of cheese cloth, and conidia were quantified with a Spencer Bright-Line hemocytometer (American Optical Company, Buffalo, NY, USA).” (Lines 155–157)

**Line 165. incidence ‘on’ plants not ‘experienced by’**

We changed this text:

“To evaluate disease incidence on plants across the *Microstegium* density gradient…” (Line 204)

**Line 239. (Fig. 4C) not (Fig. 4B)**

We changed this text:

“…peaking at intermediate densities when grown with *Eragrostis* (Fig 4C).” (Line 295)

**Line 301. ‘do not’ rather than ‘don’t’**

We changed this text:

“Future efforts that aim to better characterize host-pathogen interactions between *Microstegium* or native plant species and *Bipolaris* fungi could confirm infection by attempting to re-isolate the fungus after inoculation.” (Lines 454–456)

**Line 308. sufficient to ‘shift plant’ community not ‘plant shift’**

We changed this text:

“However, whether disease-induced changes in growth of plant species, as observed in our study, are sufficient to shift plant community structure is an important area of research [22].” (Lines 404–406)
